# Improving stability in two-dimensional transistors with amorphous gate oxides by Fermi-level tuning

**DOI:** 10.1038/s41928-022-00768-0

**Published:** 2022-06-02

**Authors:** Theresia Knobloch, Burkay Uzlu, Yury Yu. Illarionov, Zhenxing Wang, Martin Otto, Lado Filipovic, Michael Waltl, Daniel Neumaier, Max C. Lemme, Tibor Grasser

**Affiliations:** 1 Institute for Microelectronics, TU Wien, Vienna, Austria; 2 AMO GmbH, Aachen, Germany; 3 Chair of Electronic Devices, RWTH Aachen University, Aachen, Germany; 4 Ioffe Institute, Saint Petersburg, Russia; 5 Christian Doppler Laboratory for Single-Defect Spectroscopy in Semiconductor Devices at the Institute for Microelectronics, TU Wien, Vienna, Austria; 6 Chair of Smart Sensor Systems, University of Wuppertal, Wuppertal, Germany

**Keywords:** Electronic properties and devices, Two-dimensional materials, Electrical and electronic engineering, Electronic devices, Electronic devices

## Abstract

Electronic devices based on two-dimensional semiconductors suffer from limited electrical stability because charge carriers originating from the semiconductors interact with defects in the surrounding insulators. In field-effect transistors, the resulting trapped charges can lead to large hysteresis and device drifts, particularly when common amorphous gate oxides (such as silicon or hafnium dioxide) are used, hindering stable circuit operation. Here, we show that device stability in graphene-based field-effect transistors with amorphous gate oxides can be improved by Fermi-level tuning. We deliberately tune the Fermi level of the channel to maximize the energy distance between the charge carriers in the channel and the defect bands in the amorphous aluminium gate oxide. Charge trapping is highly sensitive to the energetic alignment of the Fermi level of the channel with the defect band in the insulator, and thus, our approach minimizes the amount of electrically active border traps without the need to reduce the total number of traps in the insulator.

## Abstract (web summary)

The stability of graphene-based field-effect transistors with amorphous aluminium oxide serving as the top-gate oxide can be improved by tuning the Fermilevel of the two-dimensional channel material such that it maximizes the energy distance between the charge carriers in the channel and the defect bands in the gate oxide.

## Main

Two-dimensional (2D) semiconductors are a potential channel material for ultimately scaled field-effect transistors (FETs)^1^. In contrast to silicon, 2D semiconductors retain sizable mobilities at atomic layer thicknesses below 1 nm, a thickness that also helps to suppress short-channel effects in FETs and thus allows for physical channel lengths below 5 nm (ref. ^2^). Furthermore, the integration of 2D materials in van der Waals heterostructures provides design options for energy-efficient transistors that can overcome the limitations of thermal charge-carrier injection. In addition, 2D materials are of potential use in a broad range of applications, including photonics and optoelectronics^3^, neuromorphic computing^4^, nanoelectromechanical systems^5^, and gas and biological sensors^6^.

There is, however, currently a lack of low-resistive contacts to 2D semiconductors, which minimize the prevalent Schottky barriers^7^. In addition, there is a lack of suitable gate insulators^8^ that can ensure high interface quality, scalability^9^ and a minimum of electrically active border traps, the presence of which in the insulator close to the channel limits device stability. These two challenges are major obstacles for the industrial applications of 2D-based nanoelectronics and, being independent from each other, need to be separately addressed. There has been recent progress regarding the formation of low-barrier contacts for 2D semiconductors using contact gating^10^ or semimetallic bismuth contacts to achieve ultralow contact resistances^11^. However, the need to find a suitable insulator with a minimum number of electrically active traps remains. Stability studies of 2D FETs typically show a stability that is at least two orders of magnitude worse^12,13^ compared with silicon-based FETs^14,15^.

Measurements of FET stability typically evaluate the hysteresis in the transfer characteristics^16^ and stability of threshold voltage under prolonged periods of applied elevated gate biases and temperatures (bias temperature instability (BTI))^14^. Charge trapping inside the gate oxide has been identified as the root cause of BTI^15,17^. At elevated gate biases and temperatures, charges are transferred between the channel and gate oxide in a phonon-mediated transition^18^, with charging time constants spanning a wide range, from picoseconds to years^2,19^. Border traps in the gate oxide close to the channel determine the long-term stability and reliability of silicon FETs^15^, whereas in 2D-material-based FETs, they typically limit device stability on much shorter timescales^20^.

In amorphous oxides, the defect trap levels vary due to the differing surroundings of every instance of atomic defects^18^. In the first approximation, the energy levels of defects follow a normal distribution around the average defect levels, forming defect bands^21^. Consequently, the overall density of border traps and the widths of the corresponding defect bands can be considerably reduced by using crystalline insulators, such as hexagonal boron nitride (hBN) or calcium fluoride^8^ (CaF_2_). However, these insulators are difficult to synthesize and have several technological challenges. For example, current state-of-the-art crystalline hBN can be grown only at temperatures above 1,200 °C (ref. ^22^) and CaF_2_ requires a crystalline silicon(111) substrate for growth, allowing only back-gated configurations^23^. In addition, hBN is unsuitable for use as a scaled gate insulator because of its small dielectric constant^9^.

In this Article, we show that the stability and reliability of 2D-material-based FETs with amorphous gate oxides can be improved by tuning the Fermi level (*E*_F_) of the 2D channel material such that it maximizes the energy distance between the charge carriers in the channel and the defect bands in the gate insulator during device operation. This can be achieved via a careful selection of the 2D material and amorphous gate oxide, as well as by doping the 2D layer to shift *E*_F_ away from the defect bands in the gate insulator. Graphene FETs (GFETs) with aluminium oxide (Al_2_O_3_) as the top-gate oxide are measured and compared, where one of the device batches uses a p-doped graphene layer. The GFET batch where *E*_F_ is tuned away from the Al_2_O_3_ defect band edge shows reduced hysteresis and BTI. We further verify the approach with technology computer-aided design (TCAD) simulations^24^. In addition, double-gated GFETs are fabricated, where the back gate is used to directly tune the Fermi level in the graphene monolayer via electrostatic doping. This shows that the stability observed for top-gate operation strongly depends on the location of *E*_F_. Our approach aims to design a metal–oxide–semiconductor (MOS) system with a minimal amount of electrically active border traps without the need to reduce the total number of traps in the insulator.

## Fermi-level tuning for increasing stability of 2D FETs

**Fig. 1:**
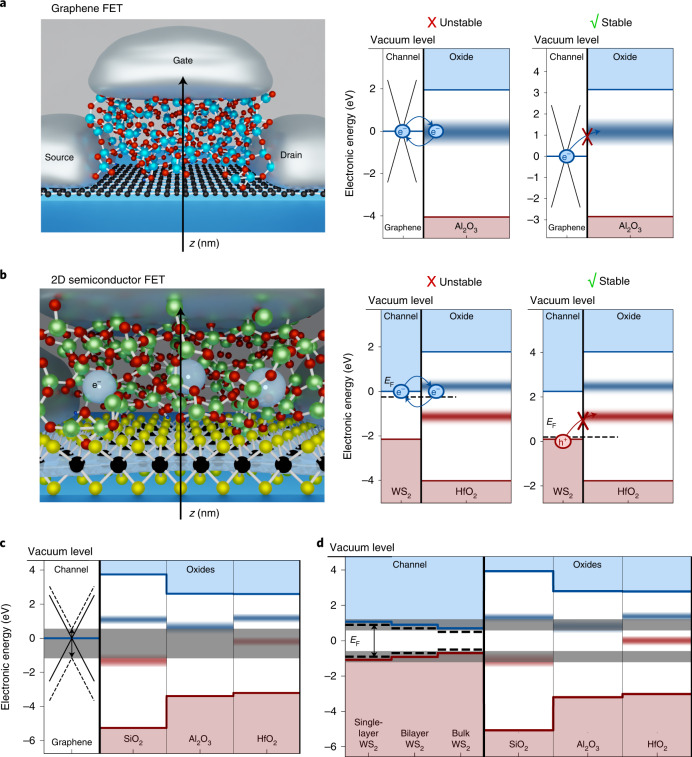
Fermi-level tuning to maximize the energetic distance to oxide defect bands. **a**, Schematic (left) shows a top-gated GFET with an Al_2_O_3_ gate oxide. For a cut through the GFET along the indicated arrow, the energetic alignment of the Fermi level to the defect band in the aluminium gate oxide is shown. In the band diagram (left), the device is electrically unstable with respect to variations in the threshold voltage as the Fermi level is aligned within the defect band. In the band diagram (right), the Fermi level has been shifted downwards, rendering the device more stable. **b**, Schematic of the charge transfer of electrons flowing through the WS_2_ channel to traps in the HfO_2_ gate oxide (left). This situation is depicted in the left band diagram where the Fermi level is aligned close to the conduction band edge, rendering the device unstable. If the Fermi level is instead aligned close to the valence band edge, the FET is stable. **c**, In this band diagram, the possible range of the graphene Fermi levels, which is currently achievable by doping, is shown as a grey-shaded region. The Fermi level can be continuously tuned within this region. **d**, Injection of electrons and holes from the band edges of WS_2_. In a layered semiconductor, the number of layers modifies the bandgap and doping determines whether electrons or holes will be the majority carriers and thus govern device stability.

Our stability-based design approach is centred on the analysis and design of the band diagram of the MOS system, including the defect bands in the insulator. Fig. 1a shows a top-gated GFET that forms an example MOS system out of aluminium (metal), Al_2_O_3_ (oxide) and graphene (semiconductor). The corresponding band diagram of a cut through the MOS stack (Fig. 1a, left, indicated by arrow) is shown on the right. Every material is characterized in this view by its electron affinity and thus the energetic distance of the conduction band edge to the vacuum level, as well as its bandgap. In the case of metals and semi-metals, the work function, the energetic distance of *E*_F_ to the vacuum level, determines the energetic location of charge carriers. In this regard, we use the Schottky–Mott rule to determine the band alignments shown as a zero-order approximation, thereby neglecting interface-specific reactions and charge imbalances that would lead to additional shifts on the order of a few hundred millielectronvolts^25^. Knowledge of the energetic position of the oxide’s defect bands and alignment to *E*_F_ is the core of our design approach.

The energetic position of defect bands in amorphous oxides is an intrinsic material property^26,27^, as defect bands are related to certain defective atomic configurations inside the amorphous material, which result in slightly varying trap levels depending on the local surroundings of the defects. In effect, the superposition of the trap levels of many atomic defects forms the defect band, characterized by the average energetic trap level $\overline{{E}_{{{{\rm{T}}}}}}$ and the standard deviation of the trap level distribution ${\sigma }_{{{{{{E}}}}}_{{{{\rm{T}}}}}}$. To experimentally determine the energetic location of the defect bands, the oxide defect states can be probed by electrical measurements, which analyse conductance variations in MOS systems^28,29^, or by electron paramagnetic resonance measurements, which detect the magnetic moment of unpaired electrons^30^. Defect bands can be theoretically determined using ab initio calculations where possible defect states and their prevalence are analysed, thereby identifying electrically active defect configurations such as oxygen vacancies^31^ or hydrogen-related defects^32^. Currently, the energetic locations of oxide defect bands are known for amorphous SiO_2_ (ref. ^29^), HfO_2_ (refs. ^26,31^) and Al_2_O_3_ (ref. ^28^) insulators. Besides the location of the defect bands, another essential property of insulator traps is their extremely broad distribution of time constants, ranging from the picoseconds regime up to years^2,19^. As these insulator traps lead to noise, hysteresis and drifts, it is important to thoroughly characterize both traps’ time constants and their energetic location within the defect bands.

Based on the band alignment of the graphene work function to the defect bands in Al_2_O_3_, we can predict the electrical stability of the threshold voltage in these FETs. In Fig. 1a (left band diagram), the work function of graphene is shown to be at 3.9 eV, which corresponds to n-doped graphene^33^. This graphene layer’s *E*_F_ lies in the middle of the Al_2_O_3_ defect band. Due to this alignment within the defect band, charge traps in the oxide capture and frequently emit charges. As the applied gate voltage modifies the charging probabilities of the defects according to the electric field^19^, *V*_TH_ depends on the biasing history and a pronounced hysteresis is visible. In addition, *V*_TH_ drifts considerably during prolonged periods of applied gate biases.

However, the theoretical considerations of Fig. 1a suggest that FET stability can be tuned by moving *E*_F_ down by p doping the graphene layer. Here the *E*_F_ value of graphene of 5.1 eV can be achieved through p doping^34^. As the graphene Fermi level is located below the Al_2_O_3_ defect band, charge transfer is unlikely. Therefore, the oxide defects are electrically inactive, resulting in stable *V*_TH_ throughout device operation, independent of the applied biases. In graphene, doping with different adsorbates and substrates results in a quasi-continuous variation in the Fermi level between 3.4 and 5.1 eV (refs. ^35,36^), which can be used to tune the Fermi level during device design to minimize the impact of oxide defect bands.

With some adaptation, the same stability-based design process can be applied to enhance the stability in FETs based on 2D semiconductors; Fig. 1b shows the schematic of a WS_2_ FET with HfO_2_ top-gate oxide. If the Fermi level is aligned close to the conduction band, electrons within WS_2_ are the majority charge carriers dominating the current flow in Schottky-barrier FETs^7^. As the conduction band edge of WS_2_ is aligned with the electron-trapping defect band of HfO_2_, charge transfer to oxide defects is frequent. If WS_2_ was p doped instead of n doped, holes at the valence band edge would be the majority (Fig. 1b, right band diagram). As the valence band edge of WS_2_ is located below the hole-trapping band in HfO_2_, the charging of oxide defects is highly unlikely. Therefore, for p-doped WS_2_ in combination with HfO_2_ gate oxide, there are no electrically active oxide traps, leading to a stable *V*_TH_ during device operation. It should be noted that for 2D semiconductors, the charges are always injected from the conduction or valence band edges. Thus, when designing a stable n-type or p-type MOSFET, a suitable combination of 2D semiconductor and oxide needs to be selected. In this context, a recent study has suggested that Fermi-level pinning in MoS_2_ is weaker if the oxide defect bands can be avoided. It was observed that the degree of Fermi-level pinning is reduced when using Al_2_O_3_, which possesses no oxide defect band in the vicinity of the valence band edge of MoS_2_, instead of SiO_2_ in back-gated FETs^37^.

The physical possibilities for tuning the stability in the context of stability-aware device design are illustrated in Fig. 1c,d. By doping the graphene layer, graphene’s *E*_F_ can be tuned within the entire grey-shaded area (Fig. 1c). Thus, the design freedom for a stability-based device is large in graphene FETs; furthermore, the role of SiO_2_ defect bands can be reduced with an *E*_F_ alignment in the middle of the two defect bands, whereas the impact of the Al_2_O_3_ defect band can be minimized when using p-doped graphene layers. For 2D semiconductors like WS_2_, the freedom for stability-aware design is smaller. Fig. 1d shows that either the conduction or valence band edge can be chosen via doping. However, n-type WS_2_ presumably will be electrically unstable for the amorphous oxides investigated here, whereas stable p-type FETs could be designed using Al_2_O_3_ or HfO_2_.

It is worth noting that several studies have reported high densities of fixed charges at the interfaces of 2D materials with amorphous oxides, for example, MoS_2_/SiO_2_ (refs. ^25,38^), WS_2_/SiO_2_ (ref. ^39^) or graphene/SiO_2_ (ref. ^40^). This evidence suggests that there might be a loss of charge neutrality at the ill-defined interfaces between van der Waals–bonded 2D layers and amorphous oxides, causing deviations from the Schottky–Mott rule^25^. These deviations result in offsets to the band alignments that can be determined, for example, with internal photoemission measurements^41^ or scanning probe techniques^42^. Although these offsets would need to be taken into account for optimum matching of the Fermi level at a maximum distance to the oxide defect bands, they are neglected for the proof-of-concept study presented here.

At the same time, the intentional placement of charges at the interface could be used to shift the band edges away from the oxide defect bands using, for example, surface charge transfer doping^43^. However, fixed charges at the interfaces would also degrade the mobility in the semiconducting 2D channel^25^. This could be avoided by using more complex gate stacks with electric dipoles at the interfaces between different oxides. Such a dipole engineering approach has been successfully used to improve the reliability of silicon FETs with an HfO_2_/SiO_2_ gate stack^21,44^.

**Fig. 2:**
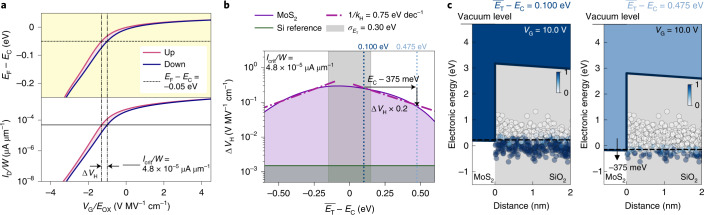
Estimation of stability improvement based on TCAD simulations. **a**, Calculated distance of the MoS_2_ Fermi level to its conduction band edge (top). The hysteresis width Δ*V*_H_ is extracted at the threshold voltage, defined as *E*_F_ being located 50 meV below the conduction band edge. From the simulated transfer characteristics of the MoS_2_ FETs based on SiO_2_ (bottom), the constant-current criterion of *I*_crit_ = 4.8 × 10^−5^ μA μm^−1^ was used to evaluate Δ*V*_H_. **b**, Hysteresis width Δ*V*_H_ is shown on a logarithmic scale as a function of the distance of the oxide trap level $\overline{{E}_{{{{\rm{T}}}}}}$ to the MoS_2_ conduction band edge *E*_C_. If *E*_C_ is moved 375 meV down, away from the trap band, the hysteresis width improves by a factor of 5. **c**, At two different locations of *E*_C_, namely, at $\overline{{E}_{{{{\rm{T}}}}}}-{E}_{{{{\rm{C}}}}}$ = 0.100 eV in dark blue and 0.475 eV in light blue corresponding to the colours of the dotted lines in **b**, the band diagrams of the MoS_2_/SiO_2_ system are shown, demonstrating how fewer oxide traps change their charge state if the conduction band edge is shifted down, leading to a reduction in the hysteresis width by half an order of magnitude.

**Extended Data Fig. 1:**
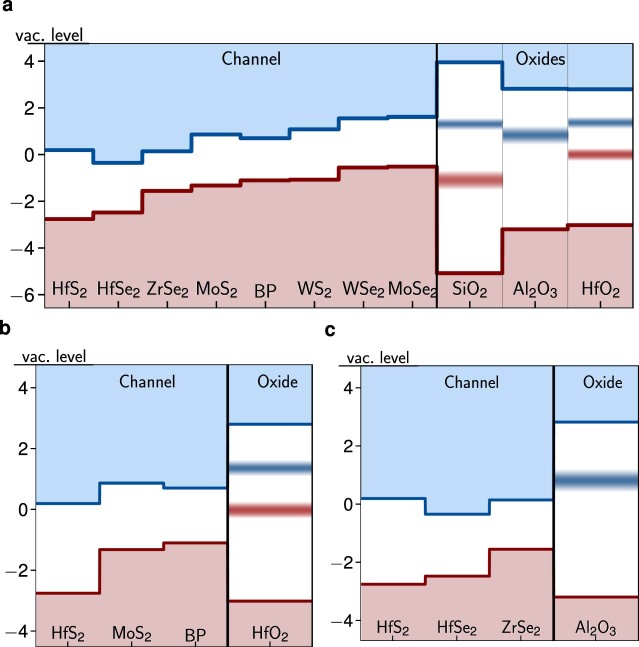
Band alignment of various 2D semiconductors with amorphous oxides. Band diagram showing the band alignment of several monolayer 2D semiconductors to the three most common gate oxides and their respective defect bands. In (a) the band edge alignment of the 2D semiconductors is shown together with the defect bands in three oxides to give an overview over possible combinations. Based on (a) potentially stable combinations of 2D semiconductors with HfO_2_ are shown in (b), revealing BP as a promising candidate. In (c) the selection for Al_2_O_3_ is presented, where HfSe_2_ or ZrSe_2_ could maximize the distances of conduction and valence band edges to the defect band.

To estimate the electrical stability improvement that can be achieved by Fermi-level tuning in FETs with amorphous oxides, we simulated the hysteresis width in FETs based on 2D semiconductors in relation to the location of the conduction band edge, *E*_CB_. For simulations, we used the previously developed drift-diffusion-based TCAD methodology^45^ coupled to a non-radiative multiphonon model^18^ (Supplementary Section 1). In Fig. 2, we calculated the hysteresis width in a model system of monolayer MoS_2_ with a back-gate oxide of SiO_2_ (ref. ^45^). We evaluated the hysteresis width at *V*_TH_, defined here as the voltage where the Fermi level is located –0.05 eV below the conduction band edge (Fig. 2a). Based on the criterion for *E*_F_ − *E*_CB_, a constant-current criterion was defined and the hysteresis width was evaluated as a function of varying distance of the trap level $\overline{{E}_{{{{\rm{T}}}}}}$ to *E*_CB_. For an oxide defect-band width of ${\sigma }_{{{{{{E}}}}}_{{{{\rm{T}}}}}}$ = 0.3 eV, the hysteresis width can be reduced by half an order of magnitude if the conduction band edge is shifted 350 meV downwards, as illustrated in the band diagrams in Fig. 2c. These shifts in the conduction or valence band edges can be achieved, for example, by transitioning from monolayers to bulk material (Fig. 1d). For example, in WS_2_, the conduction and valence band edges shift by approximately 160 meV when using bilayers instead of monolayers, or by about 370 meV when using bulk WS_2_ (ref. ^46^). Thus, we would expect that n-type WS_2_ FETs with an HfO_2_ gate oxide are more stable when using bulk WS_2_ as a channel compared with thinner WS_2_ layers. In cases where an ultimately thin monolayer channel is required, electrically stable FETs could be designed by choosing a different combination of 2D semiconductor and insulator. For example, increased electrical stability is predicted for BP/HfO_2_ FETs and for ZrSe_2_/Al_2_O_3_ FETs (Extended Data Fig. 1 and Supplementary Section 2).

**Extended Data Fig. 2:**
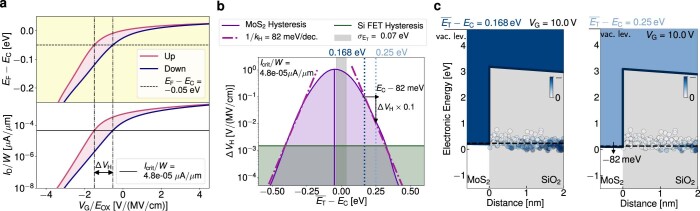
TCAD estimate of the stability improvement for insulators with narrow defect bands. For a defect band which is only 0.07eV wide, the hysteresis can be considerably reduced with Fermi level tuning. Here, a model system of MoS_2_ and SiO_2_ with a hypothetical, narrow defect band is used. (a) The hysteresis width Δ*V*_H_ is extracted at the threshold voltage, defined as *E*_F_ being located at 50 meV below the conduction band edge, see the bending of the band edges at the top. This corresponds to a constant current criterion of *I*_crit_ = 4.8 × 10^−5^*μ*A/*μ*m. (b) The hysteresis width Δ*V*_H_ is shown as a function of the distance of the oxide trap level E_T_ to the MoS_2_ conduction band edge *E*_C_. If *E*_C_ is moved 82 meV away from the trap band, the hysteresis width will improve by one order of magnitude. (c) At two different locations of *E*_C_, namely at *E*_T_ − *E*_C_ = 0.168 eV in dark blue and 0.25 eV in light blue corresponding to the colors of the dotted lines in (b), the band diagrams of the MoS_2_/SiO_2_ system are shown, demonstrating how fewer oxide traps change their charge state if the conduction band edge is shifted down.

It should be noted that for narrower defect bands, the improvement accessible by tuning the semiconductor band edges is much larger. For example, we repeated the calculations shown in Fig. 2 for an insulator defect band of only 0.07 eV. In this example, the hysteresis width is reduced by one order of magnitude by shifting the conduction band edge by 82 meV (Extended Data Fig. 2 and Supplementary Section 3). Such a reduction in the widths of the defect bands is expected for crystalline gate insulators, such as hBN or CaF_2_ (ref. ^8^). Independently, graphene, with its continuous tunability of *E*_F_ over an interval of nearly 2 eV, provides the largest design freedom. Due to the possibility to tune the Fermi level in graphene by a few 100 meV through moderate doping, we chose graphene/Al_2_O_3_ as a model system to experimentally verify our stability-based design approach.

## Graphene Fermi level and Al_2_O_3_ defect bands

**Fig. 3:**
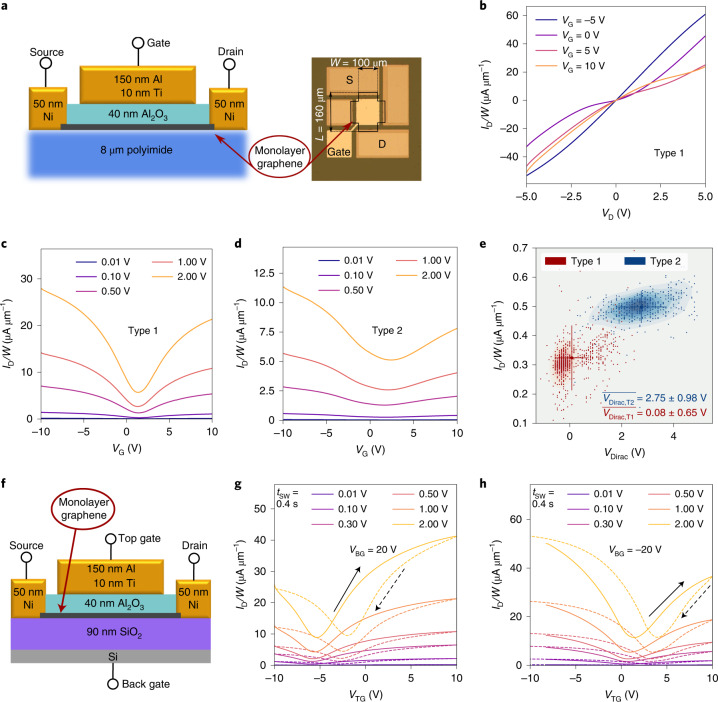
GFET design and performance. **a**, Schematic showing the cross section (left), and the optical microscopy image shows the top view of the device layout (right). S, source; D, drain. **b**,**c**, Output (*I*_D_–*V*_D_) characteristics of a representative device of Type 1 (**b**) and transfer (*I*_D_–*V*_G_) characteristics (**c**). Type 2 GFETs have the same layout as Type 1 GFETs, but are based on a different CVD-grown graphene layer from another vendor. **d**, Transfer (*I*_D_–*V*_G_) characteristics of a representative device based on Type 2 graphene. Here only the forward sweeps are shown; the backward sweeps are reported in Fig. 5. **e**, Mean current and voltage at the Dirac point are compared for the Type 1 and Type 2 devices (*V*_Dirac,T1_ and *V*_Dirac,T2_, respectively). These values were calculated from the transfer characteristics measured on 50 different GFETs of every type using *V*_D_ = 0.1 V, with every single measurement value given as a small dot. The large dots show the mean value and the bars denote the error bars, giving an error of 1*σ*. **f**, Cross section through the GFETs with a silicon wafer as the global back gate. **g**,**h**, For these double-gated GFETs, *I*_D_–*V*_TG_ curves recorded at *t*_SW_ = 0.4 s are shown for *V*_BG_ = 20 V (**g**) and *V*_BG_ = –20 V (**h**) including the backward sweep (dashed lines).

**Extended Data Fig. 3:**
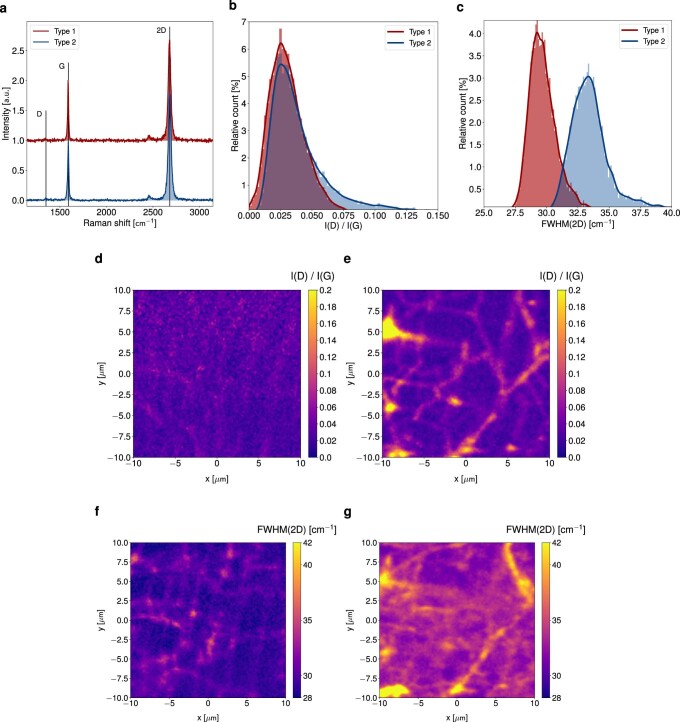
Raman spectroscopy on Type 1 and Type 2 Graphene. In Fig. (a), the median Raman spectra with respect to the intensity ratio I(D)/I(G) are shown for Type 1 and Type 2 Graphene. Fig. (b) shows the distribution of the relative count of ratios I(D)/I(G) for different locations across the surface, showing a more defective tail of locations for Type 2. In Fig. (c) the distribution of the FWHM of the 2D peak of the Raman spectra is compared for both graphene types. Fig. (d) shows a spatial map of the intensity peak ratio I(D)/I(G) calculated from the measured Raman spectra of Type 1 Graphene. In Fig. (e) the same spatial map as recorded on Type 2 Graphene is shown. Both maps cover a representative area of 20 *μ*m × 20 *μ*m, the x and y axis give the location on the sample surface in *μ*m. Fig. (f) shows the spatial map of the FWHM of the 2D peak of the Raman spectra for Type 1 Graphene which can be compared with the same map for Type 2 in Fig. (g).

To test our proposed stability-based design, we fabricated two batches of GFETs using graphene samples with different doping levels, termed as Type 1 graphene and Type 2 graphene. In addition, we fabricated GFETs with a double-gated structure where the back gate can be used to electrostatically dope the graphene channel^47,48^. In the first two batches, graphene monolayers form a channel with an area of *W* × *L* = 100 μm × 160 μm on top of mechanically flexible polyimide (PI) substrates^49^ (Fig. 3a). In the top-gated device layout, a 40-nm-thick amorphous Al_2_O_3_ layer, grown by atomic layer deposition, is used as the gate oxide. The two fabricated GFET batches using Type 1 and Type 2 graphene mainly differ in the respective doping and quality of their graphene channels. These graphene layers were purchased from different vendors using different parameters for the chemical vapour deposition (CVD) process and layer transfer. Type 1 graphene exhibits a work function that results in a small distance of *E*_F_ to the Al_2_O_3_ trap band ($\overline{{E}_{{{{\rm{T}}}}}}$). According to our theory, this small value of $\overline{{E}_{{{{\rm{T}}}}}}-{E}_{{{{\rm{F}}}}}$ predicts electrically unstable devices. In contrast, Type 2 graphene is p doped with a higher distance of *E*_F_ to $\overline{{E}_{{{{\rm{T}}}}}}$, predicting electrically more stable FETs. Furthermore, the graphene films have vastly differing qualities, with Type 2 graphene exhibiting a higher concentration of defects (Extended Data Fig. 3 and Supplementary Section 4 show the respective Raman spectra). Although we anticipate that a higher defect concentration would lead to an overall degraded GFET performance, if our hypothesis is correct, the p doping of Type 2 devices should nevertheless lead to more stable devices due to the larger distance of *E*_F_ from the defect band.

To assess the functionality and performance of our GFETs, the output (*I*_D_–*V*_D_) and transfer (*I*_D_–*V*_G_) characteristics are shown for a representative Type 1 GFET (Fig. 3b,c). We observe ambipolar device operation with kinks in the output characteristics at higher *V*_D_, features typical for GFETs^47^. This local saturation of the output characteristics has been linked to a pinch-off region in the monolayer graphene channel, where the majority charge type changes and the charge concentration declines locally^47^. When we compare these characteristics with those of Type 2 graphene FETs (Fig. 3d), it is evident that the higher quality of Type 1 graphene leads to higher current densities. Based on two-probe measurements of the *I*_D_–*V*_G_ characteristics, we estimate the field-effect mobilities to reach up to 5,000 cm^2^ V^−1^ s^−1^ in Type 1 GFETs, four times the average mobility of about 600 cm^2^ V^−1^ s^−1^ in Type 2 GFETs. These results are expected based on the Raman analysis and originate from the higher amount of defects in Type 2 graphene.

**Extended Data Fig. 4:**
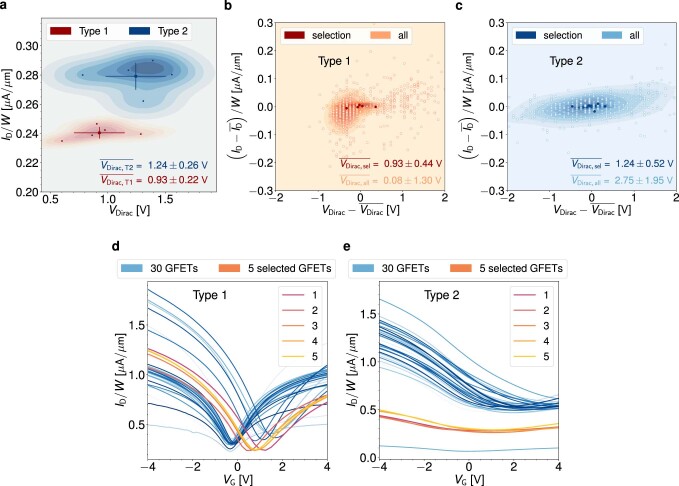
Variability of GFETs based on Type 1 and Type 2 Graphene. Here, the full statistics from Fig. 3(e) in the main manuscript are compared to the characteristics of 5 selected devices for each graphene type, selected for the hysteresis and long term stability studies. In (a) the average Dirac point location of the 5 selected devices is compared. Fig. (b) shows the variability of the 5 selected devices of Type 1 in comparison to all 50 devices of Type 1 and Fig. (c) shows the equivalent comparison for Type 2 devices. In Fig. (d), the full transfer characteristics of 30 Type 1 GFETs are compared to the transfer characteristics of the 5 selected Type 1 GFETs and in Fig. (e) the comparison for Type 2 GFETs is shown.

Negatively charged dopants in Type 2 lead to higher variability and shift *V*_Dirac_ towards more positive voltages, as evident from the comparison of *V*_Dirac_ measured on 50 devices for each graphene type (Fig. 3e). More details on the variability of the two types of GFET studied here are provided in Extended Data Fig. 4 and Supplementary Section 5. A more positive *V*_Dirac_ corresponds to a higher p doping of the sample and correlates with a higher work function (*E*_W_)^34^. Pristine graphene has a work function of 4.56 eV (ref. ^50^), which is shifted towards higher values by p doping^34^ and towards smaller values by n doping^33,35^. To calculate the Fermi-level location in the two graphene types, we obtain the charge-carrier concentration (*n*) based on the analytic expression for *n* in the MOS capacitor^36,51^. At a top-gate bias of 0 V, we extract the charge-carrier concentration (n) caused by the intrinsic doping of graphene samples

1 $$n\left({V}_{{{{\rm{TG}}}}}\right)=\frac{{C}_{{{{\rm{tot}}}}}}{q}\left|{V}_{{{{\rm{TG}}}}}-{V}_{{{{\rm{Dirac}}}}}\right|,$$

with the total gate capacitance of the structure (Ctot) and elementary charge q. Here, *C*_tot_ is given by the capacitance of Al_2_O_3_ (*C*_ox_) in series with the capacitance of 0.5 nm van der Waals gap (*C*_vdW_) and the quantum capacitance of graphene^52^ (*C*_q_), amounting to *C*_tot_ = 0.16 μF cm^−2^. This expression gives a p-doping density for Type 1 graphene of *n*_1_ = 5.5 × 10^10^ cm^−2^ and for Type 2 graphene of *n*_2_ = 2.8 × 10^12^ cm^−2^. Thus, Type 2 graphene is more p doped by an additional doping density of approximately 2.75 × 10^12^ cm^−2^. These hole densities in the graphene layers at 0 V gate voltage determine the work function via^33,42^

2 $${E}_{{{{\rm{W}}}}}=\hslash {\nu }_{{{{\rm{F}}}}}\sqrt{\uppi n},$$

where the Fermi velocity in graphene is *ν*_F_ = 1.1 × 10^6^ m s^−1^ (ref. ^53^). Consequently, we obtain *E*_W1_ of Type 1 graphene to be 4.6 eV and *E*_W2_ of Type 2 graphene to be 4.8 eV, that is 0.2 eV higher (Supplementary Section 6 shows the calculation of the work function). For all the typical FET metrics, Type 2 graphene suggests poorer performance, including lower mobility and lower ON/OFF ratio. However, because *E*_W2_ is higher than *E*_W1_, our stability-based design theory suggests that Type 2 graphene should produce more stable GFETs, which is what we set out to prove below.

**Extended Data Fig. 5:**
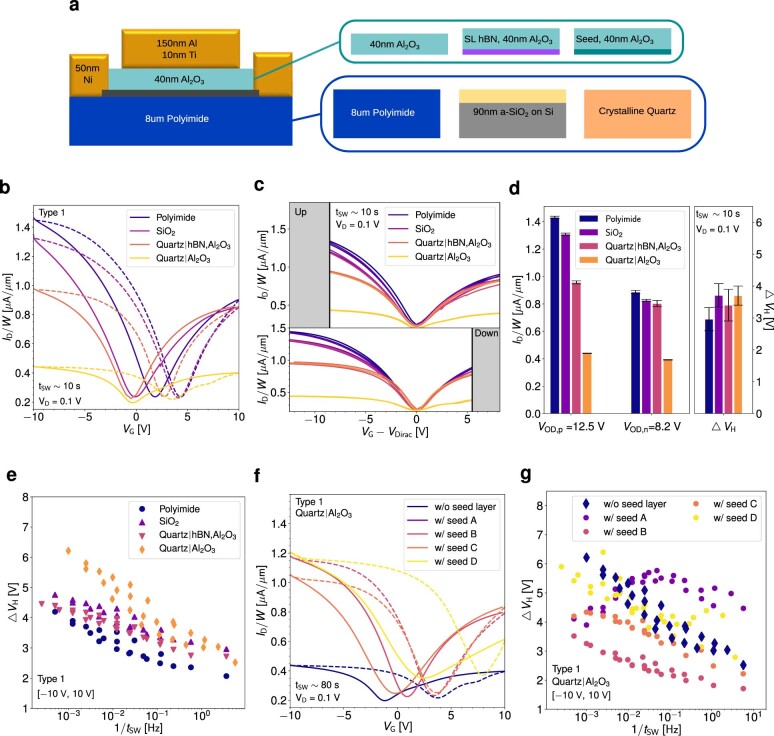
Impact of the substrate choice and the gate oxide to channel interface on the hysteresis. The impact of the variation of the substrate from flexible polyimide to SiO_2_ grown on Si, to quartz wafers is studied. For the devices on quartz in one batch a single layer of hBN was used as an interlayer between graphene and Al_2_O_3_, see the overview in (a). In (b) the hysteresis in the *I*_D_*-V*_G_ on various substrates is compared. In (c) the curves are shifted by their respective Dirac point (*I*_D_ − (*V*_G_ − *V*_Dirac_)) to allow for a better comparison of the overdrive current, the currents at a certain overdrive voltage above the Dirac point. Three measurements of every device type are shown. In (d) the currents at fixed overdrive voltages and hysteresis widths are compared for three measurements on every device type. In (e) the dependence of the hysteresis width on the sweep time for the different substrates is shown. Fig. (f) shows the impact of the seed layer for the growth of the Al_2_O_3_ top gate insulator. As the quality of the seed layer varies substantially across the wafer, measurements were repeated for different devices at different locations across the wafer, represented by A, B, C and D. In (g) the hysteresis widths on the GFETs with seed layer are compared to a GFET without seed layer.

To further analyse our model system, we fabricated devices with Type 1 graphene but using thermal SiO_2_ on silicon and quartz substrates instead of a flexible PI layer. In addition, the quality of the interface between graphene and Al_2_O_3_ was modified by transferring single-layer CVD-grown hBN layers before the ALD deposition or by sputtering ~2-nm-thick aluminium as a seed layer for the Al_2_O_3_ growth process. As shown in Extended Data Fig. 5 and Supplementary Section 7, the substrate primarily impacts the maximum current density, whereas the quality of the interface with Al_2_O_3_ impacts device stability.

Furthermore, we fabricated double-gated GFETs using 90 nm SiO_2_ as a back-gate oxide and the silicon wafer as a global back gate (Fig. 3f). This configuration allows electrostatic control of the doping of the monolayer graphene channel via the back gate^48^. By applying a positive voltage at the back gate of, for example, 20 V (Fig. 3g), the Dirac voltage of the top gate is shifted towards more negative voltages, corresponding to a smaller work function of graphene. Conversely, *V*_BG_ = –20 V makes *V*_Dirac_ of the top gate more positive and results in a higher graphene work function (Fig. 3h). Consequently, these devices are expected to be more electrically stable at higher negative *V*_BG_ than at higher positive *V*_BG_, as shown below.

**Fig. 4:**
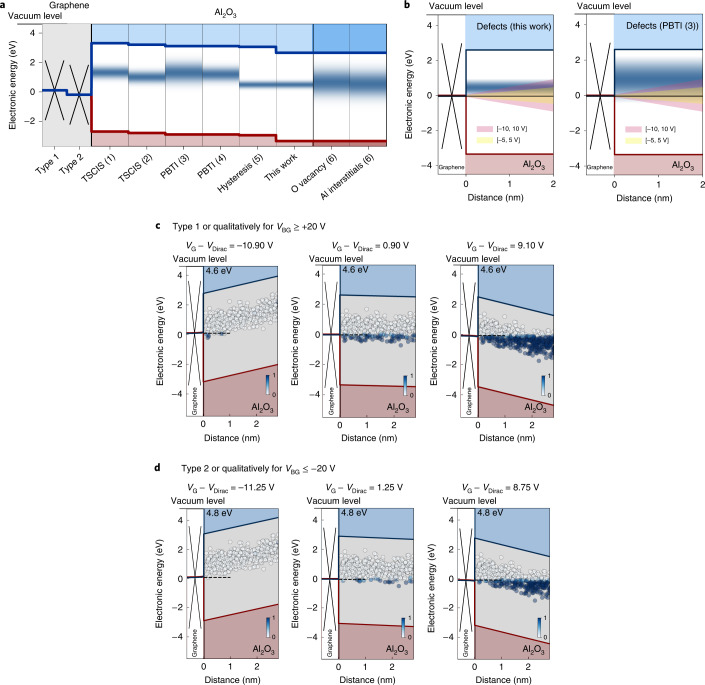
Defect band alignment in Al_2_O_3_. **a**, Band diagram illustrates the alignment of the Al_2_O_3_ defect band to Type 1 and Type 2 graphene. The location of defect bands as extracted from experiments is shown: (1) (ref. ^28^), (2) (ref. ^54^), (3) (ref. ^55^), (4) (ref. ^56^) and (5) (ref. ^20^). Also, the alignment of the defect band caused by oxygen vacancies and Al interstitials in amorphous Al_2_O_3_ is shown according to DFT calculations (6) (ref. ^57^). **b**, Active region probed by measurements in the [–5, 5 V] and [–10, 10 V] range is shown for two defect band alignments for Type 1 GFETs. **c**, Schematic of the band diagrams showing the charging and discharging of defects in Al_2_O_3_ for Type 1 graphene with a work function of *E*_W_ = 4.6 eV—a value that can be qualitatively reached also with *V*_BG_ ≥ +20 V. **d**, Band diagrams for Type 2 graphene with *E*_W_ = 4.8 eV are shown, an effective doping level qualitatively accessible with *V*_BG_ ≤ –20 V.

To accurately determine the alignment of *E*_F_ in graphene to the electron-trapping band of the amorphous Al_2_O_3_ gate oxide at $\overline{{E}_{{{{\mathrm{T}}}}}}$, knowing the precise location of the oxide defect band is essential. Several studies have investigated the alignment of this defect band using trap spectroscopy by charge injection and sensing (TSCIS)^28,54^, BTI^55,56^ and hysteresis measurements^20^. The defect band alignments of Al_2_O_3_ as obtained from the literature are shown in Fig. 4a, with the corresponding parameters listed in Supplementary Section 8. Based on density functional theory (DFT) calculations, this defect band can be associated with either oxygen vacancies^57^ or aluminium interstitials^57^. For our study, we use a normally distributed defect band with the mean defect level at *E*_C_ − *E*_T_ = 2.15 ± 0.30 eV below the conduction band edge of Al_2_O_3_. The electron affinity (*χ*) of Al_2_O_3_, which determines the location of the conduction band edge, varies in the literature. Here we use 1.96 eV, as obtained from internal photoemission measurements^41^. For all the measurement ranges used in our work, we only probe the lower part of a potentially wider defect band further up, as reported using other methods^55^. This is illustrated in Fig. 4b, where the regions that can be probed by measurements are shaded in red and yellow. These shaded regions reach the upper edge of the defect band used here, but cover only the lower part of the wider defect band reported elsewhere^55^. For Type 1 graphene, *E*_F_ is aligned within the defect band (small $\overline{{E}_{{{{\rm{T}}}}}}-{E}_{{{{\rm{F}}}}}$, electrically unstable) (Fig. 4c), whereas for Type 2 graphene, it is aligned below the defect band (high $\overline{{E}_{{{{\rm{T}}}}}}-{E}_{{{{\rm{F}}}}}$, electrically stable) (Fig. 4d). Below, we discuss that as proposed above, the 200 meV downward shift of the Fermi level of Type 2 graphene is sufficient to make the *V*_Dirac_ value of these GFETs more electrically stable.

## Hysteresis dynamics of GFETs

**Fig. 5:**
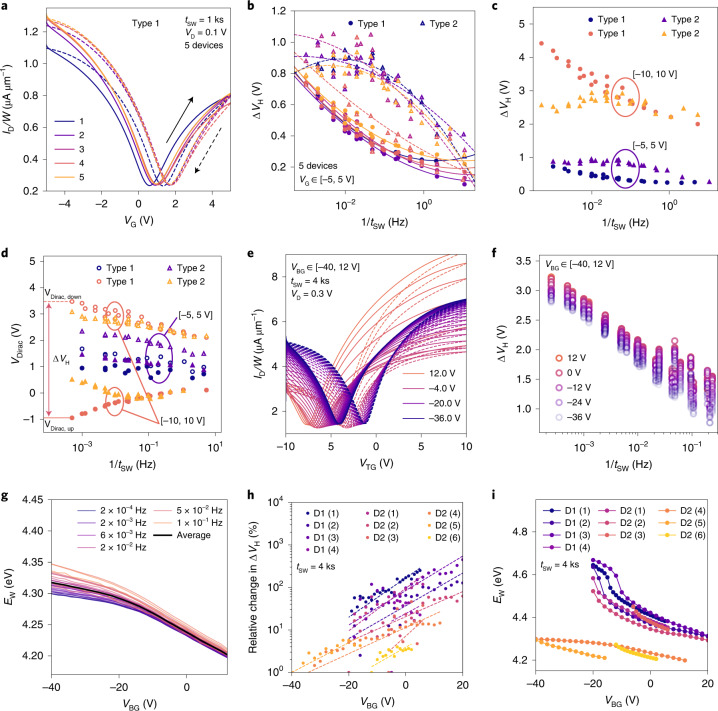
Hysteresis in the transfer characteristics of GFETs. **a**, Hysteresis in the transfer characteristic measured on five different Type 1 GFETs, illustrating the variability of the devices. **b**, Hysteresis width as a function of 1/*t*_SW_ is shown for five different devices for each graphene type. The circles and triangles are used to represent Type 1 and Type 2 GFETs, respectively, whereas the solid and dashed lines are guides to the eye for Type 1 and Type 2 GFETs, respectively. **c**, Hysteresis width for Type 1 and Type 2 GFETs is shown as measured on one representative GFET per type. **d**, Dirac-point shifts of the up and down sweeps are shown with empty/full symbols for *V*_Dirac,down_/*V*_Dirac,up_, respectively. **e**,**f**, For double-gated GFETs, the *I*_D_–*V*_G_ curves at slow sweeps are compared for two different *V*_BG_ values (**e**) with the respective hysteresis widths as a function of the inverse sweep time (**f**). **g**, Extent of doping the GFET as a function of the back gate voltage, for various sweep times, based on *V*_Dirac_ and equations (1) and (2). **h**, Relative change in the hysteresis width for the slowest sweeps is shown for different measurement rounds on two GFETs (D1 and D2) at varying *V*_BG_ values, illustrating that the hysteresis width increases for higher *V*_BG_. **i**, Respective effective electrostatic doping for the measurement rounds on D1 and D2.

We first compare the double-sweep transfer characteristics for a small voltage range of [–5, 5 V] on five GFETs based on Type 1 graphene (Fig. 5a). We note little variability, which is confirmed when studying the hysteresis width Δ*V*_H_ as a function of the inverse sweep time (*t*_SW_), namely, the sweep frequency (*f* = 1/*t*_SW_). In Fig. 5b, the hysteresis width as a function of the sweep frequency is shown for five GFETs based on Type 1 graphene and five GFETs based on Type 2 graphene. Type 2 devices show a considerably higher variability of Δ*V*_H_ than Type 1 devices, which is linked to the increased variability of *V*_Dirac_ on Type 2 (Fig. 3e). In addition, on Type 2 GFETs, the hysteresis is higher; for both types, the largest hysteresis is observed for the slowest sweeps as the largest number of oxide defects can change their charge state^20^. Since the observed hysteresis critically depends on the voltage ranges used for the gate-voltage sweeps, we compare the bias ranges used with ranges for various applications (Supplementary Section 9), concluding that the gate-oxide fields investigated here are standard operating conditions for radio-frequency applications.

An increased bias range of [–10, 10 V] increases the hysteresis, because more oxide defects become accessible for charge transfer (Fig. 5c) for the representative Type 1 and Type 2 GFETs. To shed more light on this behaviour, the dynamics of the Dirac voltage shifts are analysed as a function of the sweep frequency (Fig. 5c). For the [–5, 5 V] sweep, *V*_Dirac,up_ and *V*_Dirac,down_ as a function of the sweep frequency show similar slopes for both types. However, for the 10 V sweep range and Type 1 GFET, *V*_Dirac,up_ is shifted to more negative voltages in slow sweeps, whereas *V*_Dirac,down_ is shifted to more positive voltages. This indicates that for large sweep ranges on Type 1 GFETs, a large amount of electrons are emitted from the oxide traps between –10 V and *V*_Dirac,up_, whereas for Type 2 GFETs, charge trapping can be neglected in this interval. This reversed drift of *V*_Dirac,up_ to more negative voltages at slower sweeps results in an increase in the hysteresis width in Type 1 GFETs (Fig. 5c). The increased hysteresis at large sweep ranges for Type 1 GFETs confirms our hypothesis that as the *E*_F_ value of Type 1 GFET is located closer to the Al_2_O_3_ defect band, the GFETs are electrically less stable.

The band alignments shown in Fig. 4c,d qualitatively explain the larger hysteresis in Type 1 GFETs compared with Type 2: in Type 1 GFETs biased at *V*_Dirac_, a considerable number of defects are negatively charged. If a negative voltage is applied, these defects discharge due to band bending, and thus, *V*_Dirac_ is shifted to more negative voltages during a slow up-sweep (Fig. 5c). In contrast, in Type 2 GFETs, the Fermi level is located below the defect band at *V*_Dirac_, as its Fermi level has been shifted down by 200 meV via p doping. Thus, most defects are neutral at the Dirac voltage. If a long time is spent with the GFET biased at negative voltages, the charge states do not change and the location of *V*_Dirac_ during the up-sweep is stable, independent of the sweep time.

**Extended Data Fig. 6:**
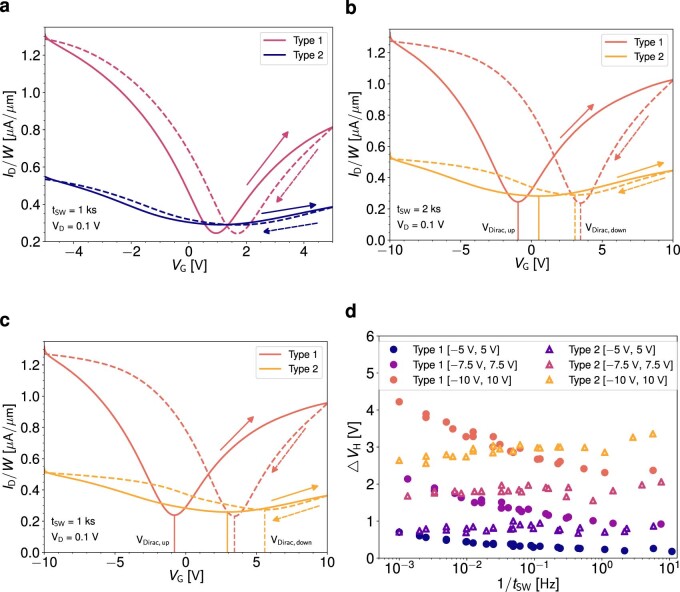
Hysteresis in Type 1 and Type 2 GFETs for varying devices and sweep ranges. In (a) the hysteresis on devices with two different graphene types is compared for the sweep voltage range of [-5V, 5V]. The same hysteresis comparison for a larger sweep voltage range of [-10V, 10V] is shown in (b). The hysteresis as measured on two different devices of Type 1 and Type 2 for the same large sweep voltage range of [-10V, 10V] is given in (c). On the devices where the characteristics are shown in (c) the hysteresis width as a function of different sweep voltage ranges and sweep times are compared in (d).

In summary, the higher $\overline{{E}_{{{{\rm{T}}}}}}-{E}_{{{{\rm{F}}}}}$ of Type 2 graphene with respect to the Al_2_O_3_ defect band leads to a smaller hysteresis width for large sweep ranges. At small gate-bias ranges and fast hysteresis sweeps, Type 2 devices suffer from more charge trapping at the defective interface with the Al_2_O_3_ insulator, and the hysteresis is similar or even higher in Type 2 devices compared with Type 1 devices (Fig. 5b, Extended Data Fig. 6 and Supplementary Section 10). For fast sweeps, fast traps at the defective interface in Type 2 GFETs increase the hysteresis, giving the impression of a frequency-independent hysteresis width (Fig. 5c). Type 1 GFETs exhibit a cleaner interface but a smaller $\overline{{E}_{{{{\rm{T}}}}}}-{E}_{{{{\rm{F}}}}}$ with respect to Al_2_O_3_ defects, strongly degrading the GFETs during slow sweeps. For high gate-bias ranges and slow sweeps, the border traps of Al_2_O_3_ dominate the device stability, thus more stable operation of Type 2 GFETs is observed.

In the double-gated configuration, the graphene layer can be dynamically doped in situ (Fig. 3f). To determine the impact of electrostatic back-gate doping on the top-gate stability, we characterized the hysteresis in the top-gate *I*_D_ (*V*_TG_) curves after biasing the devices at a static *V*_BG_. Subsequently, the top-gate hysteresis was measured at different sweep rates. In Fig. 5e, the hysteresis at the top gate is shown for slow sweeps and various back-gate voltages from 12 V down to –40 V. When comparing the hysteresis widths as a function of the sweep time and the applied back-gate voltage (Fig. 5f), two trends are clearly observed. First, the hysteresis is reduced for fast sweeps; second, the hysteresis is the smallest for the most negative *V*_BG_. It is expected that hysteresis can be reduced for high negative back-gate voltages, as the work function of the graphene channel is the highest at 4.3 eV for a higher negative *V*_BG_ (Fig. 5g). The work function was calculated based on the measured *V*_Dirac_ as a function of *V*_BG_ and equations (1) and (2). At high graphene work functions, the Fermi level is located closer towards the lower edge of the defect band in the Al_2_O_3_ top-gate oxide, reducing the number of charge-trapping events.

**Extended Data Fig. 7:**
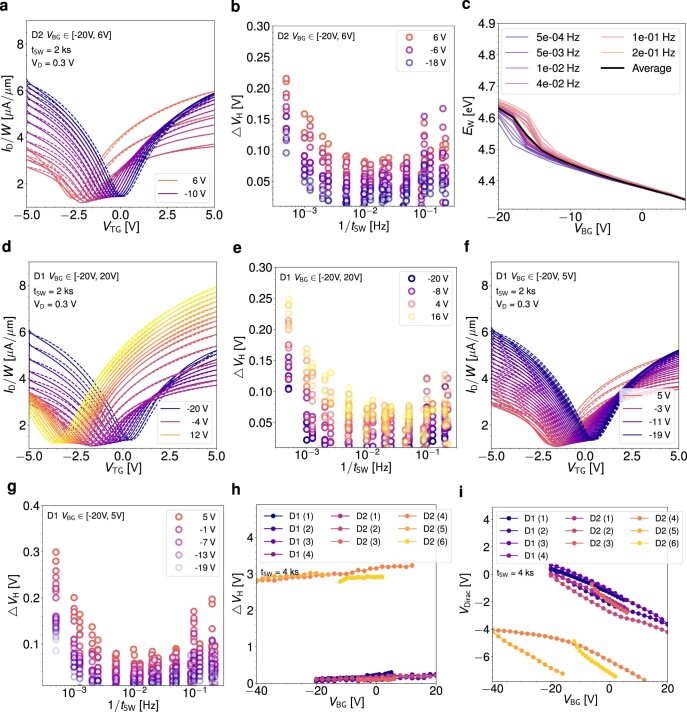
Hysteresis in the transfer characteristics of double gated GFETs. In (a) the *I*_D_(*V*_G_) curves for top gate sweeps within ± 5 V are shown for *V*_BG_ varying between 6V in the first measurement and -20V in the last measurement on GFET D2 in round (1). In (b) the hysteresis width for this measurement round (D2(1)) for varying sweep times is shown and in (c) the work function shift as a function of the applied back gate voltage is compared for all the sweep frequencies. In Fig. (d), the measurement round (2) on D1 with *V*_TG_ ∈ ± 5V and *V*_BG_ ∈ [-20V, 20V] is shown at the slowest sweep time and the hysteresis width for varying *t*_SW_ is shown in (e). Figs. (f) and (g) show similar graphs for the measurement round (1) on D1 with *V*_TG_ ∈ ± 5V and *V*_BG_ ∈ [-20V, 5V]. Fig. (h) shows the absolute hysteresis width for different measurement rounds as a function of *V*_BG_ and Fig. (i) shows the dependence of *V*_Dirac_ on *V*_BG_ which is used to calculate *E*_W_.

In Fig. 5h, we compare the relative change in Δ*V*_H_ over different measurement rounds on two double-gated GFETs, namely, D1 and D2 (Extended Data Fig. 7 and Supplementary Section 11). Throughout these ten measurements, there is an exponential dependence of Δ*V*_H_ on the applied back-gate voltage, as expected from our theoretical calculations (Fig. 2b). An improvement of a factor of up to 4.5 is observed for a work-function shift of 340 meV, as shown in the corresponding comparison of the work function for these measurements (Fig. 5i). Thus, we observe an improvement of about 750 meV dec^−1^ when more negative back-gate voltages are applied, in good agreement with the theoretical results (Fig. 2b). However, in this double-gated configuration, full improvement cannot be achieved in every measurement round; in particular, for high *V*_BG_, a strong modulation of the work function by the back gate is hindered by the charging of oxide traps in SiO_2_. Nevertheless, all the hysteresis measurements show that a shift in the graphene work function to higher values, away from the defect band in Al_2_O_3_, successfully reduces the amount of electrically active border traps, thereby stabilizing the GFETs.

## Stability under static gate bias

**Fig. 6:**
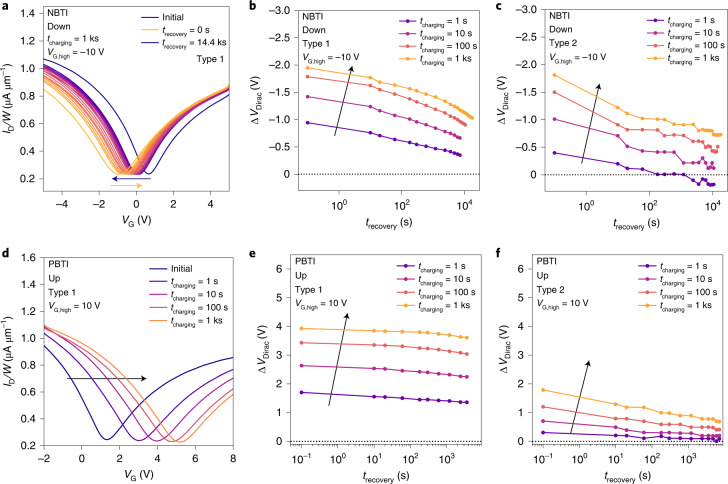
Long-term electrical stability assessed using BTI. In BTI measurements, the FET is subjected to extended periods of elevated gate bias and the drifts in the Dirac voltage during the degradation and recovery periods are recorded. **a**, Type 1 device subjected to –10 V for 1 ks. **b**,**c**, Type 1 FET is subjected for increasing time spans to elevated NBTI gate bias of –10 V (**b**), resulting in a larger degradation than observed for the same conditions on Type 2 FETs (**c**). **d**–**f**, When applying an elevated PBTI voltage level of 10 V to the GFETs (**d**), the Dirac voltage of Type 1 devices drifts more and barely recovers (**e**) compared with their Type 2 counterparts (**f**). To avoid an impact of the measurement history, the measurements shown were performed on different devices.

**Extended Data Fig. 8:**
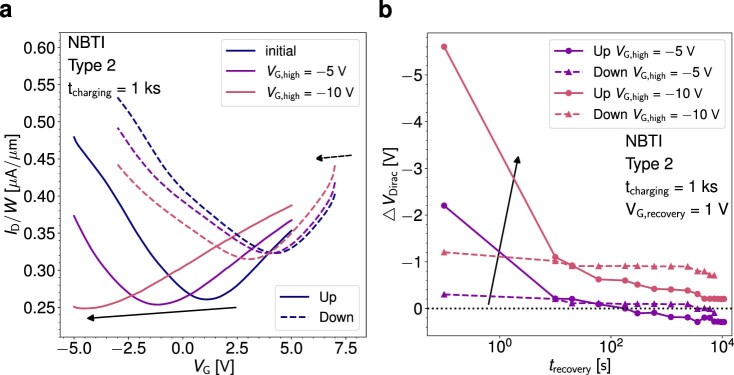
NBTI evaluation using up-sweeps. In (a) the degradation on Type 2 devices is compared when evaluated using up sweeps in comparison to down sweeps. In (b) it can be seen that when using up sweeps for the evaluation of NBTI degradation a fast trapping component is active which also increases the over-recovery towards more positive Dirac voltages.

To evaluate the long-term stability of GFETs, we analysed the Dirac-voltage shifts (Δ*V*_Dirac_) after static elevated gate voltages (*V*_G,high_) were applied for varying charging times (*t*_charging_). We record the magnitude of the initial Δ*V*_Dirac_ shift and monitor the recovery after the increased gate-biasing period with fast *I*_D_ (*V*_G_) sweeps at logarithmically spaced recovery times. In Fig. 6a, the fast *I*_D_ (*V*_G_) sweeps recorded during the recovery from negative gate biasing (negative-bias temperature instability (NBTI)) at –10 V are shown. Such BTI measurements give a complementary perspective on the long-term stability and reliability of FETs compared with the hysteresis measurements discussed earlier. Although during hysteresis measurements, GFETs are subjected to slow up and down gate-voltage sweeps, during a BTI measurement, an elevated gate bias is applied for a certain charging time and the recovery of the Dirac point is recorded. With this well-established measurement scheme, the impact of border traps is studied^12,13,15^. Thus, in a BTI measurement, the observed hysteresis during the probing sweeps is small and not in focus. To avoid measurement artefacts coming from fast traps causing the hysteresis in the probing *I*_D_ (*V*_G_) curves, the down-sweep *I*_D_ (*V*_G_) curves are used to evaluate the *V*_Dirac_ shifts for all NBTI measurements and the up-sweep *I*_D_ (*V*_G_) curves for positive gate biasing (positive-bias temperature instability (PBTI)) (Methods and Extended Data Fig. 8).

**Extended Data Fig. 9:**
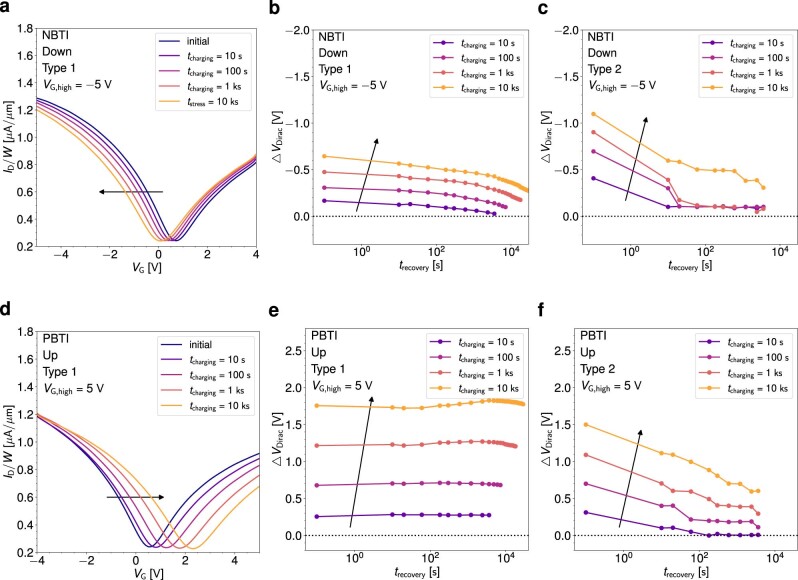
BTI for a reduced high gate bias level of 5V. In (a) the fast sweep *I*_D_(*V*_G_) curves after negative bias at -5V for increasingly long charging times are shown on Type 1 devices. The corresponding recovery traces can be seen in (b) for Type 1 and in (c) for Type 2. In an analog way, in (d) the fast sweep *I*_D_-*V*_G_ curves after positive bias of 5 V are shown. For Type 1 devices the degradation is barely recoverable (e). On Type 2 FETs the degradation is mostly recoverable also for long charging times, see (f).

**Extended Data Fig. 10:**
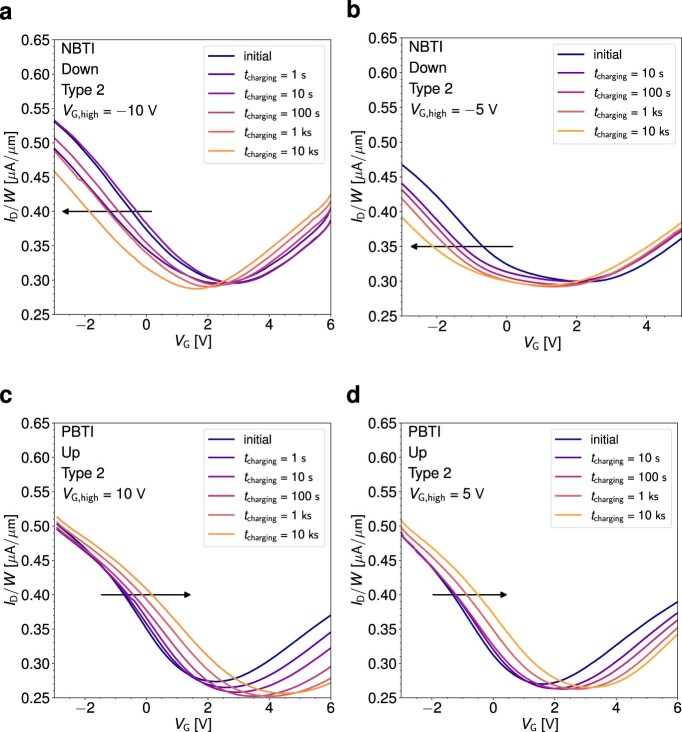
BTI on Type 2 GFETs. For Type 2 GFETs the fast sweep *I*_D_(*V*_G_) curves after positive and negative gate biasing are shown. These transfer characteristics were recorded to evaluate *V*_Dirac_ directly after the biasing at an elevated gate voltage, at *t*_recovery_ = 0.5 s. In (a), NBTI curves are shown at −10 V, and in (b), at −5 V. In (c) and (d), PBTI curves are shown at 10 V and 5 V, respectively. The corresponding fast sweep *I*_D_−*V*_G_ curves for Type 1 GFETs are shown in Figs. 6(a) and (d), and in Extended Data Figs. 9(a) and (d).

NBTI measured by subjecting the devices to a gate bias of –10 V for increasingly long charging times is shown for Type 1 GFETs (Fig. 6b) and for Type 2 GFETs (Fig. 6c). As planned, the *V*_Dirac_ shifts are smaller on Type 2 devices than on Type 1 devices. GFETs based on Type 2 graphene are more stable with respect to long-term degradation because the graphene *E*_F_ is further away from the Al_2_O_3_ defect band (Fig. 4d). Therefore, on Type 2 GFETs, fewer oxide traps change their charge state during negative gate biasing, resulting in smaller shifts in *V*_Dirac_, which also recover faster as the traps that emit electrons are located closer to the interface and thus have smaller time constants. Extended Data Fig. 9 shows the recovery traces of NBTI at –5 V. For Type 2 GFETs, slight over-recovery^13^ is observed for the shortest charging time of 1 s (Supplementary Section 12). This over-recovery is also visible for the fast *I*_D_ (*V*_G_) sweeps used for the BTI evaluation of Type 2 GFETs (Extended Data Fig. 10). In Fig. 6d, the fast *I*_D_ (*V*_G_) sweeps measured after a positive bias at 10 V are shown, together with the corresponding recovery traces for Type 1 GFETs (Fig. 6e) and Type 2 GFETs (Fig. 6f). For both device types, degradation on applying positive biases (PBTI) are higher than NBTI shifts, as the Fermi level in graphene is at the lower edge of the Al_2_O_3_ defect band (Fig. 4c). Thus, the number of defects that become more negatively charged during a positive bias is larger than the number of defects that emit one of their electrons during a negative bias. As Type 2 graphene is more p doped, *E*_F_ is located further away from the Al_2_O_3_ defect band, ultimately reducing the amount of charge trapping without the need to modify the insulator or reduce the total number of traps.

Interestingly, throughout the charging times, the shifts on Type 1 devices do not recover, whereas the shifts on Type 2 devices show complete recovery, even for a short charging time of 1 s. This observation was confirmed when subjecting the devices to a smaller gate-bias voltage of 5 V (Extended Data Fig. 9). We hypothesize the active creation of defects in Al_2_O_3_ to explain the permanent component of BTI degradation in Type 1 GFETs. In silicon FETs using SiO_2_ as a gate dielectric, the permanent component of BTI has been associated with gate-sided hydrogen release^58^. We speculate that a similar mechanism of bias-facilitated oxide defect creation in Al_2_O_3_ is responsible for the permanent PBTI observed for our GFETs, which will need to be investigated by future studies.

## Conclusions

We have reported an approach to improve the electrical stability of FETs based on 2D materials. Charge trapping at the border traps in amorphous oxides is the principal cause of the threshold-voltage drifts and reduced long-term stability in 2D FETs. Therefore, the impact of defect bands in amorphous gate oxides can be reduced by tuning the energy alignment of the Fermi level. We demonstrate our approach using GFETs with Al_2_O_3_ as the top-gate oxide and two different types of graphene, which differ in their doping and Fermi-level alignments based on their respective fabrication methods. Our measurements show that the GFETs, which are based on the more p-doped Type 2 graphene with a higher (*E*_T_ − *E*_F_), have a smaller hysteresis and increased stability of their Dirac voltage when subject to prolonged elevated gate biases. Furthermore, by electrostatic doping of the graphene channel via a back gate, the hysteresis width can be reduced by a factor of up to 4.5. These results suggest that more stable and reliable 2D-material-based FETs using common amorphous gate oxides can be built by minimizing the impact of defect bands in the gate oxides during design.

In 2D semiconductors, the design options mainly consist of choosing suitable materials depending on n or p doping, or varying the thickness of the channel material. There is more design freedom with graphene, as the graphene Fermi level can be tuned over a range of up to 2 eV. Moreover, our approach to improve stability may be universally applicable to other insulators, such as crystalline insulators, where the impact of narrow insulator-defect bands can be reduced further than in amorphous oxides^8^. However, future studies will be necessary to clarify what levels of stability can be achieved by Fermi-level tuning in systems based on amorphous oxides and crystalline insulators. In addition, the stability-based design approach relies on prior knowledge about the energy location of the defect bands in the oxide, which at the moment is incomplete.

## Methods

### Device fabrication

Our top-gated GFETs were fabricated on spin-coated PI substrates using photolithography. First, the flexible substrate was prepared by spin coating PI in the liquid form on a Si wafer and subsequently curing the layer. The thickness of the solidified PI film was about 8 μm. During the fabrication process, a rigid Si substrate was used as a support layer. In the next step, a CVD-grown graphene layer was transferred to the PI substrate. We study two batches of GFETs where the channel is formed by graphene samples purchased from different vendors, namely, vendor 1 (Type 1) and vendor 2 (Type 2). For Type 1 devices, CVD graphene was transferred from the copper growth substrate using a polymethyl methacrylate (PMMA)-assisted wet transfer method^59^; for Type 2 GFETs, the transfer was performed by vendor 2. The Type 1 graphene flake covered an area of 2 × 2 cm^2^ and was of higher quality than the Type 2 flake (which covered a six-inch wafer). The different qualities of the graphene layer were confirmed by Raman spectroscopy (Extended Data Fig. 3 and Supplementary Section 4). The graphene layer was patterned in an oxygen-plasma etch step to form channels with length (*L*) of 160 μm and width (*W*) of 100 μm. In the next step, the source and drain contacts were deposited by sputtering 50 nm Ni, followed by a lift-off process. This step was followed by growing 40 nm Al_2_O_3_ with atomic layer deposition (ALD) on top of the devices to form the gate oxide in a top-gated configuration. To finalize the GFETs, the top-gate electrode was fabricated by sputtering 10 nm Ti and 150 nm Al and patterned in a lift-off process. To be able to contact the source and drain pads, vias were opened through the Al_2_O_3_ with a wet-buffered oxide etchant. For our double-gated GFETs, we transferred CVD graphene monolayers from vendor 1 using a PMMA-assisted wet transfer method^59^ to a 90 nm SiO_2_ on a Si wafer. Subsequently, the graphene layer was patterned in an oxygen-plasma etch step to form channels of *L* = 80 μm and *W* = 50 μm and the source and drain contacts were deposited by sputtering 50 nm Ni followed by a lift-off process. After this step, the gate oxide of 40 nm Al_2_O_3_ was grown using ALD and the top-gate electrode was deposited with a sputter process.

### Measurement technique

Our electrical measurements were performed in a vacuum at room temperature and in complete darkness. The devices were examined with the PI supported on a silicon wafer. From two-probe measurements, we extracted the field-effect mobility of the GFETs and found it to be 4,000 cm^2^ V^−1^ s^−1^ for Type 1 graphene and 1,000 cm^2^ V^−1^ s^−1^ for Type 2 graphene. The Hall mobility of both the samples was found to be slightly higher. The hysteresis was analysed by measuring the double-sweep *I*_D_–*V*_G_ characteristics using different sweep times *t*_SW_ and sweep ranges of ${V}_{{{{\rm{Gmin}}}}}$ and ${V}_{{{{\rm{Gmax}}}}}$. The hysteresis width Δ*V*_H_ was extracted as the difference between the forward- and reverse-sweep *V*_Dirac_ value. As suggested in our previous work^20^, we expressed the hysteresis dynamics using Δ*V*_H_ (1/*t*_SW_) traces. Finally, the BTI degradation/recovery dynamics were analysed using subsequent degradation/recovery rounds with either fixed stress time *t*_deg_ and increasing high-voltage levels *V*_G,high_, or fixed *V*_G,high_ and increasing *t*_deg_. During the recovery period, we applied a constant recovery voltage of *V*_G,recovery_ = 1 V between the sweeps. This voltage is chosen to be close to the charge-carrier equilibrium at *V*_Dirac_. To avoid artefacts from fast traps charged during the sweep, the down-sweep *I*_D_–*V*_G_ curve is used to monitor the recovery of NBTI^13^. The characteristics obtained when using up-sweeps to measure the NBTI recovery are shown in Extended Data Fig. 8. For PBTI measurements, the recording of the up-sweep minimizes artefacts^13^; thus, we used *I*_D_–*V*_G_ sweeps from negative to positive voltages for the evaluation of PBTI. As was suggested in our previous study on GFETs^13^, we expressed the BTI degradation magnitude using a Dirac-point voltage shift Δ*V*_Dirac_ and plotted it versus the relaxation time *t*_r_. To gain more statistics, all our measurements were repeated on several devices.

## Supplementary information

Supplementary Information Supplementary Sections 1–12, Figs. 1–3 and Table 1.

## Data Availability

The data that support the findings of this study are available from the corresponding author upon reasonable request.

## Code availability

For the calculations of the hysteresis width depending on the defect band alignment, simulations of the transfer characteristics of GFETs and calculations of charge trapping in the oxide defect bands, we used GTS Minimos-NT (https://www.globaltcad.com/products/gts-minimos-nt/) and the implementation of non-radiative multiphonon model therein^24^. For educational purposes and academic research, this software can be used free of charge via an online Web interface (https://www.globaltcad.com/simonline/). For the central conclusions relevant to this paper, the simulations could be performed using the Comphy code, which is publicly available from https://comphy.eu/ (ref. ^26^).
