# Supplementary material for: Improving stability in two-dimensional transistors with amorphous gate oxides by Fermi-level tuning
Source: Nat Electron. 2022 Jun 2;5(6):356–66. doi: 10.1038/s41928-022-00768-0 (PMC9236902; doi:10.1038/s41928-022-00768-0)
Supplement: Supplementary file 1 — Supplementary Sections 1–12, Figs. 1–3 and Table 1. [file 41928_2022_768_MOESM1_ESM.pdf]

nature portfolio

https://doi.org/10.1038/s41928-022-00768-0

Supplementary information

Improving stability in two-dimensional transistors with amorphous gate oxides by Fermi-level tuning

In the format provided by the
authors and unedited

Supplementary Information
Improving stability in two-dimensional transistors with amorphous gate oxides by Fermi-level tuning

Contents

1 TCAD modeling methodology 1
2 Band alignment for various 2D semiconductors 3
3 Stability improvement for narrow defect bands 3
4 Raman spectroscopy 3
5 Variability and device selection 4
6 Calculation of the work function 5
7 The impact of substrate and interface on the hysteresis 6
8 Details about the Al_2O_3 defect band 8
9 Operating conditions for GFETs 9
10 Hysteresis comparison for GFETs of Type 1 and 2 9
11 Additional hysteresis data for double gated GFETs 10
12 Additional measurements at static gate bias 11

1. TCAD modeling methodology

Here, we use a drift-diffusion based TCAD model to simulate the device characteristics.[1] The drift-diffusion equations are computationally very efficient and are particularly well-suited to describe the transport in micro-scale experimental devices[2] such as the large-area GFETs studied here. In order to incorporate novel materials into the simulation framework, the macroscopic material response functions, like for example the band gap, have to be adapted. For graphene, this has first been established by Ancona[3] and for 2D semiconductors this

approach was further developed to account for the layered structure[4] and charge trapping at border traps in the oxide.[5] This approach was used to estimate the impact of shifts in E_{CB} on the hysteresis width V_{H} in Figure 2 of the manuscript.

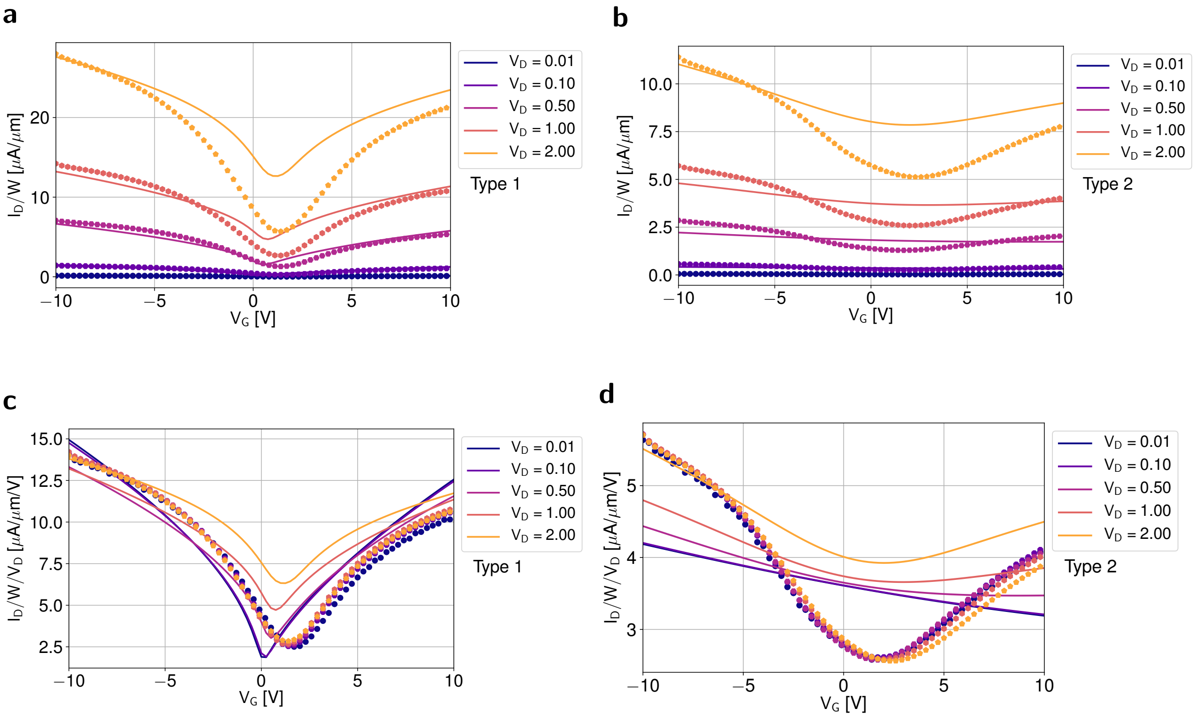

Figure S1. Modeling results for the transfer characteristics of our GFETs based on Type 1 and Type 2 graphene. The full circles represent measurement data and the solid lines the results of drift-diffusion simulations. In (a) the simulation results are shown for Type 1 GFETs and in (b) for Type 2 GFETs. The drain current is normed by the applied drain voltage (V_{D}) in (c) for Type 1 GFETs and in (d) for Type 2 GFETs. Here, the measured curves at different drain voltages fall on top of each other and the accuracy of the simulation data can be assessed more accurately.

The agreement we obtained between simulations and experimental characteristics is shown in Figure S1. In Figure S1a an acceptable agreement for Type 1 devices is shown and a reasonable agreement for Type 2 devices is shown in Figure S1b. In Figures S1c and S1c the same characteristics are shown but now normed by the applied drain voltage (V_{D}). In this way the experimental data for all transfer characteristics measured at different drain voltages fall on top of each other. The configurations of the drift-diffusion simulations shown here were used to estimate the impact of the Fermi level shift on the hysteresis width in the main manuscript in Figure 2 and Extended Data Fig. 2 and to create the band diagrams as cuts through the simulated devices in Figures 4(c) and 4(d).

2. Band alignment for various 2D semiconductors

In Extended Data Fig. 1(a) the band alignment of several 2D semiconductor monolayers is shown together with the defect bands in the three most common gate oxides according to Box 3 in Illarionov *et al.*[6] This band diagram assumes that the Schottky-Mott rule holds in a zero-order approximation[7–9] and is based on the band alignments of 2D semiconductors obtained from density functional theory using PBE functionals and the G_0W_0 method, as reported in the 2D materials database https://cmrdb.fysik.dtu.dk/c2db/.[10] Based on Extended Data Fig. 1(a) semiconductor monolayers which are expected to be the most stable in combination with HfO_2 are selected in Extended Data Fig. 1(b). While monolayer BP is the most promising candidate[11], also MoS_2,[12] or HfS_2[13] will likely result in electrically stable FETs. In this context HfS_2 FETs are of particular interest as HfO_2 is the native oxide to HfS_2, offering high quality interfaces via layer-by-layer oxidation[13]. A similar selection of semiconductor monolayers that will result in stable FETs in combination with Al_2O_3 is shown in Extended Data Fig. 1(c), revealing that HfS_2,[14] $HfSe_2$, and $ZrSe_2$[15] are promising candidates for electrically stable devices.

3. Stability improvement for narrow defect bands

For narrower defect bands in the gate insulators, the performance improvements which are accessible via Fermi level tuning are higher. Thus, TCAD simulations are used to calculate the hysteresis width in MoS_2 FETs with a SiO_2 back gate for varying locations of the conduction band edge for a reduced defect band width of only 0.07 eV, as for instance expected in crystalline insulators such as hBN and CaF_2. When comparing these results shown in Extended Data Fig. 2 with the results in Figure 2 in the main manuscript for a defect band width of 0.3 eV, it can be seen that for narrower defect bands much smaller shifts of the Fermi level can considerably enhance FET stability. In this example, a shift of the conduction band edge of 82 meV can reduce the hysteresis width by one order of magnitude, see Extended Data Fig. 2(b). Therefore, crystalline insulators which have narrower defect bands considerably increase the electrical stability of 2D material based FETs.[6]

4. Raman spectroscopy

Our Raman spectroscopy measurements were performed with a 532 nm laser at 25 mW. For each spectrum the accumulation time was 0.2 s and the spectra were recorded at 100 x magnification with a 1200 cm^{-1} diffraction grating. We recorded the Raman spectra of our

samples over an area of 10 µm×10 µm forming spatial maps. In Extended Data Fig. 3(a) Raman spectra as measured on Type 1 and Type 2 graphene are compared. These spectra are the median spectra with respect to the ratio of the intensities of the G peak at $\sim 1580\,\mathrm{cm}^{-1}$ and the D peak at $\sim 1350\,\mathrm{cm}^{-1}$.[16] This means that half of the ∼ 10000 measured spectra, which were recorded across a 20 µm× 20 µm sample area, show a higher ratio of the peaks' intensities and the other half show a smaller ratio. In fact, the ratio of the G peak and the D peak in graphene is a measure for the defect density in graphene.[16] When looking at the distribution of this ratio across the entire measured sample area of Type 1 and Type 2 graphene in Extended Data Fig. 3(b) there is an extended tail of higher ratios in Type 2 graphene, an indication for defective local areas in Type 2 graphene. The spatial distribution of the defective areas can be seen on spatial Raman maps shown in Extended Data Figs. 3(e) and (g). Another measure of the local layer quality which indicates nanometer-scaled strain variations in the graphene layer is the width of the 2D peak at $\sim 2690\,\mathrm{cm}^{-1}$,[17,18] measured as the full width half maximum (FWHM) of the peak. When comparing the distribution of the width of the 2D peak for Type 1 and Type 2 graphene in Extended Data Fig. 3(c) the larger peak width for Type 2 signifies a reduced layer quality in comparison to Type 1 graphene. This reduced layer quality, inferred from the peak width, was linked in previous works to a reduced charge carrier mobility in graphene,[19] which agrees well with the mobility assessed on the device level in the main manuscript. In the spatial map of the I(D)/I(G) ratio for Type 2 graphene in Extended Data Fig. 3(d), bright yellow regions correspond to a high ratio and in consequence a high defect density. In comparison, the spatial map for Type 1 graphene in Extended Data Fig. 3(e) does not contain any particularly defective regions, demonstrating the higher quality of Type 1 graphene. When comparing the spatial maps for the FWHM of the 2D peak of the Raman spectra in Extended Data Figs. 3(f) and (g), the higher peak width and larger variation in Type 2 also indicate a reduced layer quality for Type 2 graphene samples.

5. Variability and device selection

In Extended Data Fig. 4(a), the comparison of the Dirac point in the two GFET types is shown for 5 selected devices of Type 1 and Type 2 each. While Figure 3(e) of the main manuscript shows the full statistics of 50 devices per type, here the average Dirac point location of 5 devices per type is evaluated. These devices were fully characterized with hysteresis and BTI measurements, as shown for example in Figures 5a and 5b. As hysteresis and BTI measurements on a single device require approximately one week, it is practically impossible to characterize more than 5 devices per graphene type. Also for the selected

devices, Type 2 is clearly more p-doped than Type 1 even though the average V_{Dirac} of the selected devices are only 0.3 V apart. It should be noted that 5 devices per graphene type were selected with the intention of choosing 5 devices of similar V_{Dirac} which show a V_{Dirac} close to 0 V, as this is required to ensure that V_{Dirac} stays within the measurement window after extended applied gate biases have caused drifts. In addition, a similar V_{Dirac} guarantees similar oxide fields at the same applied $V_{\mathrm{G,high}}$. In Extended Data Fig. 4(b) and (c) the variability of the 5 selected devices which are comparatively similar are compared to the overall variation observed in the two batches. In Extended Data Fig. 4(d) and (e) the full transfer characteristics are compared for 30 GFETs and the 5 GFETs selected for the hysteresis and BTI analysis. It should be noted that a certain drift of the characteristics between the full characterization of all GFETs and the characterization of the 5 selected GFETs is expected as the full variability characterization was performed directly after device fabrication in air and the measurements on the 5 selected devices were performed during the stability analysis in vacuum several weeks later.

6. Calculation of the work function

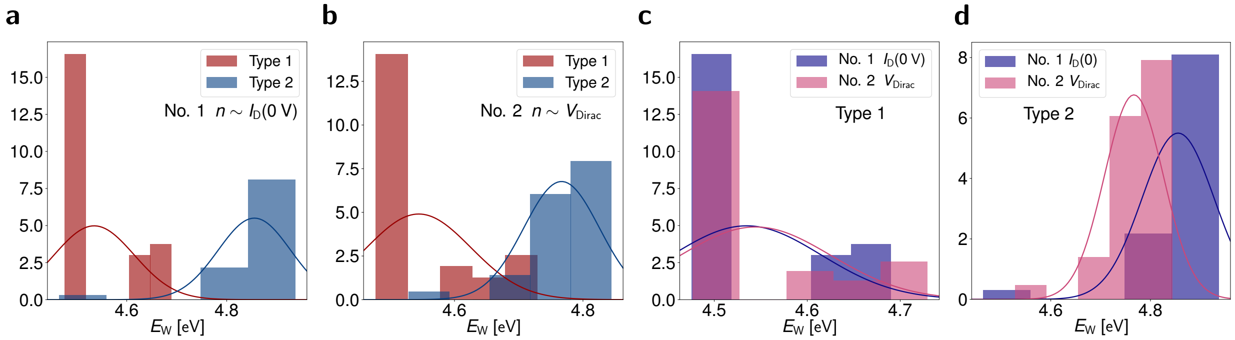

Figure S2. Comparison of the results of both calculation approaches for the work function in Type 1 and Type 2 graphene. The histograms show the distribution density (in %) of E_{W} obtained from 30 I_{D}-V_{G}s for every type. In (a) and (b) it is shown that both calculation methods for the charge density result in a stronger p-doping of Type 2 graphene. Figures (c) and (d) demonstrate that both calculation methods result in comparable E_{W} distributions.

The charge carrier density in the channel of a GFET can be evaluated on the one hand via the expression for the drain current of a MOSFET in the linear regime[20,21]

$$n\left(V_{\mathrm{G}}\right)=\frac{L}{q\mu_{\mathrm{eff}}WV_{\mathrm{D}}}I_{\mathrm{D}}\left(V_{\mathrm{G}}\right) \tag{1}$$

and on the other hand via the equation for the MOS capacitor[21–23]

$$n\left(V_{\mathrm{G}}\right)=\frac{C_{\mathrm{tot}}}{q}\left|V_{\mathrm{G}}-V_{\mathrm{Dirac}}\right|. \tag{2}$$

Both equations are evaluated for $V_{\mathrm{TG}}=0\,\mathrm{V}$, as the impact of the intrinsic doping or electrostatic doping via the back gate is investigated here. In the first equation a constant effective mobility μ_{eff} is used together with W, L, the dimensions of the GFET. In the second expression the capacitance of the device is obtained by calculating the series capacitance of the oxide capacitance $C_{\mathrm{ox}}=\varepsilon_0\varepsilon_{\mathrm{R}}/d$ of Al_2O_3, the van der Waals (vdW) capacitance of the 0.5 nm vdW gap $C_{\mathrm{vdW}}=\varepsilon_0/0.5\,\mathrm{nm}$ and the quantum capacitance of graphene that is approximated as $C_{\mathrm{q}}=2\,\mu\mathrm{F/cm^2}$.[24] In the second step the work function E_{W} of the graphene is obtained as $E_{\mathrm{W}}=\hbar\nu_{\mathrm{F}}\sqrt{\pi n}$[25–27] with the Fermi velocity of graphene $\nu_{\mathrm{F}}=1.1\times10^6\,\mathrm{ms^{-1}}$.[28] These equations were applied to 30 I_{D}-V_{G}s per device type as shown in Extended Data Fig. 4(d) and (e) to obtain the work function distribution across the wafer of one batch, shown in Figure S2. For the graphene devices of Type 1 and Type 2, there is good agreement for both calculation methods. However, it is expected that for the double gated GFETs, the neglection of the impact of the back gate voltage on the mobility and the drain current, inherent to Equation 1 will likely lead to errors. Therefore, Equation 2 is used for the double gated GFETs.

7. The impact of substrate and interface on the hysteresis

Here, the impact of variations of the substrate and of the interface between the Al_2O_3 gate oxide and the graphene channel on the electrical stability is discussed. For this purpose, four additional device batches were fabricated, all based on Type 1 graphene. For all four additional batches the graphene layer was transferred using the PMMA assisted wet transfer method.[29] One of them uses thermally grown, amorphous SiO_2 on a Si wafer as a substrate while the other three batches have been fabricated on α-quartz wafers, see Extended Data Fig. 5(a). In these three batches, the quality of the interface between the graphene and ALD Al_2O_3 was varied. On one batch Al_2O_3 was grown directly on graphene, on another batch a monolayer of hexagonal boron nitride (hBN) was transferred on top of graphene before the growth and on the last batch, a thin ($\sim 2\,\mathrm{nm}$) aluminium seed layer was sputter-deposited. This thin Al forms Al_2O_3 upon exposure to air which acts as a seed layer for the subsequent ALD growth of Al_2O_3.

Extended Data Fig. 5(b) compares the transfer characteristics of the GFETs with different interfaces. Although the current levels in our four devices are different, the hysteresis

widths are similar. For further comparison, the characteristics have been normalized to their gate overdrive ($V_G - V_{Dirac}$) for the up sweep and the down sweep, respectively, as shown in Extended Data Fig. 5(c). The average overdrive currents and hysteresis widths have been calculated for an overdrive of 12.5 V during the down sweep and an overdrive of 8.2 V during the up sweep for three measurements of each type, see Extended Data Fig. 5 (d). Best performance in terms of largest currents and smallest hysteresis widths is obtained for devices on PI, followed by devices on SiO_2. Quartz devices show the weakest performance, which is improved by the hBN interlayer underneath the ALD Al_2O_3. We hypothesize that the overdrive currents in GFETs based on various substrates differ because of scattering at defect sites on the substrate surface.[30] Similar hysteresis widths on devices based on different substrates indicate that the impact of the substrate on the hysteresis is negligible at the experimental sweep rate and bias voltage range. The hysteresis was measured and evaluated for different sweep frequencies shown in Extended Data Fig. 5(e). For sweeps from −10 V to 10 V the hysteresis width is the same for all substrate types for fast sweeps. For slow sweeps across a 20 V voltage range the hysteresis in GFETs on quartz substrates increases. We attribute this to the comparatively high surface roughness of the quartz wafer, which may lead to higher corrugations of the graphene monolayer[31] and in consequence to an inferior quality of the ALD Al_2O_3 film. In this picture, the larger observed hysteresis could be explained with a higher density of oxide defects in the amorphous Al_2O_3.

The transfer characteristics of GFETs manufactured with Al seed layers prior to Al_2O_3 ALD show a substantial variation, see Extended Data Fig. 5(f). The Dirac points, the maximum overdrive currents and the hysteresis widths vary strongly depending on the measurement location of the wafer, as shown in Extended Data Fig. 5(g). For example, the hysteresis widths for a small sweep range on location A is comparable to the hysteresis widths for the large sweep range on location B. In addition, the performance of the best devices using a seed layer is better than the performance of the devices without one, both in terms of hysteresis width and overdrive currents. Thus, the usage of a seed layer is a promising approach even though its homogeneity must be improved.

In summary, the device variations for different material systems showed that the stability of the GFETs is determined primarily by the relative alignment of graphene work function to the Al_2O_3 defect bands. At the same time, graphene is atomically thin, so that cleanliness and roughness of surrounding interfaces have an immediate impact on device performance and can degrade device stability.

8. Details about the Al_2O_3 defect band

The defect band alignment of Al_2O_3 as reported in literature[32–38] is given in Table S1.

Table S1. Parameters of the Al_2O_3 defect band. At the top, the location of defect bands as extracted from experiments is shown and at the bottom possible microscopic defect candidates calculated with DFT.

Ref./Defect	Method	Channel	Insulator	χ	E_T (E_C) in [eV]	E_T (vac)	σ_{E_T}
[32]	TSCIS	Si	SiO_2+Al_2O_3	1.3	2.0	3.3	0.5
[33]	TSCIS	Si	SiO_2+Al_2O_3	1.4	2.2	3.6	0.5
[34]	PBTI	InGaAs	Al_2O_3	1.5	1.8	3.3	0.85
[35]	PBTI	InGaAs	Al_2O_3	1.5	1.9	3.4	0.6
[36]	Hysteresis	MoS_2	Al_2O_3	1.55	2.55	4.1	0.3
our work	Hysteresis	Graphene	Al_2O_3	1.95	2.15	4.1	0.3
O vacancy[37]	DFT	-	-	1.5	1.9	3.4	-
O vacancy[38]	DFT	-	-	1.95	2.0	3.95	1.1
Al interstitial[38]	DFT	-	-	1.95	2.1	4.05	1.1

We note that the coloring of the defect band in Figures 4(c) and 4(d) in the main manuscript as an electron trapping band is a simplification. The distinction between electron trapping (acceptor-type) bands and hole trapping (donor-type) bands depends only on the overall amount of charges in the device. An acceptor-type defect changes its charge state from neutral to negative upon electron capture and a donor-type defect from positive to neutral. The only measurable difference between acceptor- and donor-type bands is the overall amount of fixed charges which is inaccessible to hysteresis and BTI measurements. Here, the coloring is based on the assumption that in the as-grown Al_2O_3 the majority of defects are neutral. Therefore, all defect bands in the upper part of the band gap are considered to be electron trapping bands and all defect bands in the lower part of the band gap are denoted hole trapping bands. Information about the charge state of the oxide defects can be obtained from DFT. According to Dicks *et al.* the thermodynamically stable states of the oxygen vacancy in amorphous Al_2O_3 are the +2 charge state and the neutral state.[38] The aluminium interstitial is stable at a charge of +3 and +1. The Al_2O_3 defect band corresponds to the transition of these two defects from their more positive to their less positive state. To explain the overall neutrality of the as-grown Al_2O_3 Dicks *et al.* invoke a balance of negatively charged Al vacancies and O interstitials and positively charged O vacancies and Al interstitials in the as-grown layer.[38] When the device is subjected to a large gate bias, the O vacancies and Al interstitials become less positively charged and in consequence the

overall charge within the system changes. The change of the net charge in this picture is the same as in the simplified image depicted in Figures 4c and 4d in the manuscript.

9. Operating conditions for GFETs

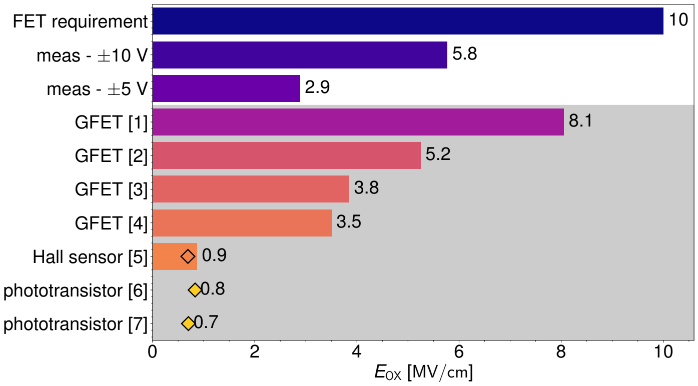

Figure S3. Here, the electrical oxide fields for measured voltage ranges are compared to voltage ranges for various applications. The field up to which GFETs should show stable operation is shown at the top in dark blue[39] and the ranges we use here are shown in purple below. Application scenarios are from [1],[40] [2],[41] [3],[42] [4],[43] [5],[44] [6],[45] [7].[46]

State-of-the-art silicon transistors operate at an electric gate field of 10 MV/cm, estimated based on the equivalent oxide thickness (EOT),[39] up to which logical switches should show stable operation. As our devices employ an aluminium oxide layer with 40 nm physical thickness as a gate oxide, their EOT amounts to $\sim$17 nm. When used in radio-frequency (RF) circuits the electric gate fields range from 3.5 MV/cm to 8.1 MV/cm[40–43] and thus the gate oxide fields we investigate here are standard operation conditions for RF applications, see also the comparison in Figure S3. If, on the other hand, GFETs are used as sensors in the form of phototransistors[45,46] or Hall elements,[44] moderate gate fields of up to 1 MV/cm are sufficient to maximize responsivity.

10. Hysteresis comparison for GFETs of Type 1 and 2

In Extended Data Fig. 6 the hysteresis in the transfer characteristics is compared on Type 1 and Type 2 devices. The comparison is performed for the small sweep range of [−5 V, 5 V] in Extended Data Fig. 6 (a) and for the large sweep range of [−10 V, 10 V] in Extended Data Fig. 6 (b). These same characteristics were measured also on another set of Type 1 and Type 2 devices. For these devices the hysteresis at a ±10 V sweep range is shown in Extended Data Fig. 6 (c). On this second set of GFETs the hysteresis was measured for three different

sweep ranges. For all sweep ranges the hysteresis width $\Delta V_{\mathrm{H}} = V_{\mathrm{Dirac,up}} - V_{\mathrm{Dirac,down}}$ was calculated for sweeps of different sweep times, see Extended Data Fig. 6 (d). As discussed in the main manuscript, the devices of Type 1 show a pronounced dependence of the hysteresis width on the sweep time with an increased hysteresis for slow sweeps. This is caused by the increased amount of oxide traps which change their charge state during the up sweep in Type 1 devices due to the more unfavorable alignment of the work function closer to the middle of the Al_2O_3 defect band. For Type 2 GFETs the hysteresis width does not depend on the sweep times which shows that defects at the more defective graphene to Al_2O_3 interface dominate the hysteresis.

11. Additional hysteresis data for double gated GFETs

In Extended Data Fig. 7 the hysteresis in the transfer characteristics is compared for various top gate sweep ranges and back gate voltages on two double gated GFETs D1 and D2. In contrast to Figures 5(e)-(g) in the main manuscript, Extended Data Figs. 7(a)-(g) were measured at a reduced sweep range of the top gate spanning $\pm 5\,\mathrm{V}$. In Extended Data Fig. 7(b) the hysteresis width is highest for the most positive V_{BG} at $6\,\mathrm{V}$ and the slowest sweep rates. The modulation of the work function as a function of V_{BG} for this measurement round is shown in Extended Data Fig. 7(c). In general, no dependence of E_{W} on the sweep frequency is expected and the small dependence that is particularly pronounced for large absolute values of V_{BG} can be associated with the charging of oxide traps in the back gate SiO_2 during the slow stepping of V_{BG}. The hysteresis widths is shown as a function of the sweep time on GFET D1 for two different measurement rounds, varying the back gate voltage between $-20\,\mathrm{V}$ and $20\,\mathrm{V}$ in Extended Data Fig. 7(e) and $6\,\mathrm{V}$ and $-20\,\mathrm{V}$ in Extended Data Fig. 7(g). These hysteresis widths consistently follow the same trends. The hysteresis is smallest for fast sweeps at the most negative V_{BG}. Thus, to directly illustrate the impact of the back gate voltage and consequently the electrostatic doping on the hysteresis width, the hysteresis width for the slowest sweep is shown as a function of the back gate voltage in Extended Data Fig. 7(h). Throughout all measurement rounds, the hysteresis width increases for an increased n-type doping of the graphene monolayer.

In the measurements performed with a larger top gate sweep range spanning $\pm 10\,\mathrm{V}$ the absolute hysteresis width is higher at about $3\,\mathrm{V}$ instead of $0.3\,\mathrm{V}$ for the smaller gate sweep range. This strong dependence on the top gate sweep range is well known as more oxide traps change their charge state between up and down sweep due to the stronger band bending.[36,47] Thus, in order to isolate the impact of the back gate and the doping on the stability, the hysteresis width is normalized to the smallest hysteresis width measured at the

most negative V_{BG}, $\Delta V_{H,normed} = (\Delta V_H - \Delta V_H(V_{BG,min}))/\Delta V_H(V_{BG,min})$. This normalized hysteresis width is shown on a logarithmic scale in the main manuscript in Figure 5(h). In addition, in Extended Data Fig. 7(i) the Dirac point shift for the different measurement rounds is shown. It becomes clear that in particular for the large back gate voltages, there is a considerable amount of charging of oxide traps in the SiO_2, as V_{Dirac} is not directly proportional to V_{BG} but instead shows a hysteresis loop depending on the measurement direction and measurement order in between different rounds. This data shown here is converted together with Equation 2 into the dependence of the work function on the back gate voltage shown in the manuscript in Figure 5(i).

12. Additional measurements at static gate bias

Measurements at static gate biases using the BTI measurement scheme[48–50] provide a complementary point of view on the degradation observed using hysteresis measurements. Whereas a comprehensive hysteresis characterization integrates over all border traps which capture charges during one sweep direction and do not emit their captured charge before the end of the reverse sweep, a BTI measurement probes the charge capture at border traps facilitated by a discrete, elevated gate bias. Thus, using BTI measurements a narrower, more well defined subset of border traps is probed.[50,51] In order to be able to separate the effects of hysteresis and BTI as clearly as possible, the impact of fast charging traps which could be visible in the fast I_D-V_G sweeps used to probe the ΔV_{Dirac} shifts is avoided by choosing the appropriate sweep direction where fast traps are discharged during the probing sweep. In Extended Data Fig. 8 the impact of the fast sweep direction on the recorded ΔV_{Dirac} shifts is shown based on the example of Type 2 devices. In Extended Data Fig. 8(a) the fast I_D-V_G sweep recorded directly after 1 ks at −5 V and −10 V respectively are shown when performed as an up sweep and as a down sweep. If an up sweep is used, the degradation is significantly increased. However, this degradation recovers very fast, as shown in Extended Data Fig. 8(b). The recovery traces extracted from the ΔV_{Dirac} shifts of up sweeps also show some over-recovery. As has been observed previously[51] this increased degradation and fast recovery is an artifact. Fast charging traps which become charged during the fast I_D-V_G sweeps are the reason for the strong degradation and fast recovery. To avoid measurement errors caused by this artifact we used for all NBTI measurements fast down sweep I_D-V_Gs to evaluate the Dirac voltage shift and for all PBTI measurements fast up sweep I_D-V_Gs.

In Extended Data Fig. 9, the degradation for increasing charging times at a constant elevated gate bias voltage of 5 V is shown. Extended Data Fig. 9(a) presents the shift of the transfer characteristics after NBTI at −5 V of Type 1 GFETs. The recovery of the NBTI

degradation at $-5\,\text{V}$ on Type 1 GFETs in Extended Data Fig. 9(b) is smaller than the recovery of the NBTI degradation of Type 2 GFETs in Extended Data Fig. 9(c). However, Type 2 GFETs recover much faster than Type 1 GFETs from the reduced voltage level at $-5\,\text{V}$, in fact for Type 2 GFETs the recovery is at 10 s nearly complete. This indicates that the fast-recovering component of the degradation could be related to an increased defect density at the interface of Type 2 graphene to the Al_2O_3 gate oxide. These fast defects have been observed in the hysteresis measurements in the hysteresis section of the main manuscript and are related to the lower quality of Type 2 graphene. In Extended Data Fig. 9(d) the transfer characteristics directly after PBTI at 5 V on Type 1 GFETs are shown. In Extended Data Figs. 9(e) and (f) the recovery traces of ΔV_{Dirac} are shown for Type 1 and Type 2 respectively. Even when 5 V are applied for 10 s the degradation does not recover on Type 1 GFETs while it recovers completely after 100 s on Type 2 GFETs.

In Extended Data Fig. 10 the fast sweep transfer characteristics used for the BTI characterization of Type 2 MOSFETs are shown. This Figure complements the I_{D}-V_{G}s of Type 1 shown in Figures 6(a) and (d) in the manuscript and in Extended Data Figs. 9(a) and (d). These characteristics show the degradation of the Type 2 GFET directly after being subjected to extended periods of elevated gate biasing at t_{recovery} =0.5 s. It can be seen that larger drifts are observed for higher electric fields, being higher in Extended Data Fig. 10(a) at $-10\,\text{V}$ than in Extended Data Fig. 10(b) at $-5\,\text{V}$ and in Extended Data Fig. 10(c) at 10 V than in Extended Data Fig. 10(d) at 5 V. In addition, PBTI leads to a more pronounced degradation than NBTI in these devices. Furthermore, the shape of the transfer characteristics in Extended Data Fig. 10(a) can provide information to understand the observed slight over-recovery for NBTI stress on Type 2 GFETs at the shortest charging time of t_{charging} =1 s, observed in Figure 6(c) in the main manuscript. If over-recovery takes place, the observed ΔV_{Dirac} shifts do not converge towards the initial state at ΔV_{Dirac} =0 V but instead overshoot with V_{Dirac} continuing to drift from negative to positive shifts. This phenomenon has been observed multiple times on FETs based on Graphene[52] and TMDs.[53,54] Here, we only observe it for the shortest NBTI charging of 1 s and thus it is probable that the over-recovery stems from the recovery voltage of 1 V (see the Methods section). These recovery conditions correspond to minor PBTI and as these PBTI drifts are stronger and less recoverable than NBTI drifts, they dominate for this trace after a recovery time of 1 ks.

References

(1) Global TCAD Solutions, *Minimos NT Manual*; 2017; Vol. 3; pp 1–305.

(2) Marin, E. G.; Perucchini, M.; Marian, D.; Iannaccone, G.; Fiori, G. Modeling of Electron Devices Based on 2-D Materials. *IEEE Transactions on Electron Devices* **2018**, *65*, 4167–4179, DOI: 10.1109/TED.2018.2854902.

(3) Ancona, M. G. Electron transport in graphene from a diffusion-drift perspective. *IEEE Transactions on Electron Devices* **2010**, *57*, 681–689, DOI: 10.1109/TED.2009.2038644.

(4) Mirabelli, G.; Hurley, P. K.; Duffy, R. Physics-based modelling of MoS_2: The layered structure concept. *Semiconductor Science and Technology* **2019**, *34*, DOI: 10.1088/1361-6641/ab121b.

(5) Knobloch, T.; Rzepa, G.; Illarionov, Y. Y.; Waltl, M.; Schanovsky, F.; Stampfer, B.; Furchi, M.; Mueller, T.; Grasser, T. A Physical Model for the Hysteresis in MoS_2 Transistors. *IEEE Journal of the Electron Devices Society* **2018**, *6*, 972–978, DOI: 10.1109/JEDS.2018.2829933.

(6) Illarionov, Y. Y.; Knobloch, T.; Jech, M.; Lanza, M.; Akinwande, D.; Vexler, M. I.; Mueller, T.; Lemme, M. C.; Fiori, G.; Schwierz, F.; Grasser, T. Insulators for 2D nanoelectronics: the gap to bridge. *Nature Communications* **2020**, *11*, DOI: 10.1038/s41467-020-16640-8.

(7) Park, S.; Schultz, T.; Shin, D.; Mutz, N.; Aljarb, A.; Kang, H. S.; Lee, C. H.; Li, L. J.; Xu, X.; Tung, V.; List-Kratochvil, E. J.; Blumstengel, S.; Amsalem, P.; Koch, N. The Schottky-Mott Rule Expanded for Two-Dimensional Semiconductors: Influence of Substrate Dielectric Screening. *ACS Nano* **2021**, *15*, 14794–14803, DOI: 10.1021/acsnano.1c04825.

(8) Afanas'ev, V. V.; Chiappe, D.; Leonhardt, A.; Houssa, M.; Huyghebaert, C.; Radu, I.; Stesmans, A. Internal Photoemission of Electrons from 2-Dimensional Semiconductors. *ECS Transactions* **2017**, *80*, 191–201, DOI: 10.1149/08001.0191ecst.

(9) Afanas'ev, V. V.; Delie, G.; Houssa, M.; Shlyakhov, I.; Stesmans, A.; Trepalin, V. Band alignment at interfaces of two-dimensional materials: Internal photoemission analysis. *Journal of Physics Condensed Matter* **2020**, *32*, DOI: 10.1088/1361-648X/ab937c.

(10) Haastrup, S.; Strange, M.; Pandey, M.; Deilmann, T.; Schmidt, P. S.; Hinsche, N. F.; Gjerding, M. N.; Torelli, D.; Larsen, P. M.; Riis-Jensen, A. C.; Gath, J.; Jacobsen, K. W.; Mortensen, J. J.; Olsen, T.; Thygesen, K. S. The Computational 2D Materials Database: High-throughput modeling and discovery of atomically thin crystals. *2D Materials* **2018**, *5*, 1–95, DOI: 10.1088/2053-1583/aacfc1.

(11) Haratipour, N.; Koester, S. J. Ambipolar Black Phosphorus MOSFETs with Record n-Channel Transconductance. *IEEE Electron Device Letters* **2016**, *37*, 103–106, DOI: 10.1109/LED.2015.2499209.

(12) Zhu, Y.; Li, Y.; Arefe, G.; Burke, R. A.; Tan, C.; Hao, Y.; Liu, X.; Liu, X.; Yoo, W. J.; Dubey, M.; Lin, Q.; Hone, J. C. Monolayer Molybdenum Disulfide

Transistors with Single-Atom-Thick Gates. *Nano Letters* **2018**, *18*, 3807–3813, DOI: 10.1021/acs.nanolett.8b01091.

(13) Lai, S.; Byeon, S.; Jang, S. K.; Lee, J.; Lee, B. H.; Park, J. H.; Kim, Y. H.; Lee, S. HfO2/HfS2 hybrid heterostructure fabricated: Via controllable chemical conversion of two-dimensional HfS2. *Nanoscale* **2018**, *10*, 18758–18766, DOI: 10.1039/c8nr06020g.

(14) Kanazawa, T.; Amemiya, T.; Ishikawa, A.; Upadhyaya, V.; Tsuruta, K.; Tanaka, T.; Miyamoto, Y. Few-layer HfS2 transistors. *Scientific Reports* **2016**, *6*, 22277, DOI: 10.1038/srep22277.

(15) Mleczko, M. J.; Zhang, C.; Lee, H. R.; Kuo, H. H.; Magyari-Köpe, B.; Moore, R. G.; Shen, Z. X.; Fisher, I. R.; Nishi, Y.; Pop, E. HfSe2 and ZrSe2: Two-dimensional semiconductors with native high-κ oxides. *Science Advances* **2017**, *3*, DOI: 10.1126/sciadv.1700481.

(16) Ferrari, A. C.; Meyer, J. C.; Scardaci, V.; Casiraghi, C.; Lazzeri, M.; Mauri, F.; Piscanec, S.; Jiang, D.; Novoselov, K. S.; Roth, S.; Geim, A. K. Raman spectrum of graphene and graphene layers. *Physical Review Letters* **2006**, *97*, 1–4, DOI: 10.1103/PhysRevLett.97.187401.

(17) Couto, N. J.; Costanzo, D.; Engels, S.; Ki, D. K.; Watanabe, K.; Taniguchi, T.; Stampfer, C.; Guinea, F.; Morpurgo, A. F. Random strain fluctuations as dominant disorder source for high-quality on-substrate graphene devices. *Physical Review X* **2014**, *4*, 1–13, DOI: 10.1103/PhysRevX.4.041019.

(18) Neumann, C.; Reichardt, S.; Venezuela, P.; Drögeler, M.; Banszerus, L.; Schmitz, M.; Watanabe, K.; Taniguchi, T.; Mauri, F.; Beschoten, B.; Rotkin, S. V.; Stampfer, C. Raman spectroscopy as probe of nanometre-scale strain variations in graphene. *Nature Communications* **2015**, *6*, 1–7, DOI: 10.1038/ncomms9429.

(19) Yamada, T.; Okigawa, Y.; Hasegawa, M.; Watanabe, K.; Taniguchi, T. Relationship between mobility and strain in CVD graphene on h -BN. *AIP Advances* **2020**, *10*, DOI: 10.1063/5.0019621.

(20) Taur, Y.; Ning, T. H. *Fundamentals of Modern VLSI Devices*, 2nd ed.; Cambridge University Press: Cambridge, 2009; pp 1–656.

(21) Wittmann, S.; Aumer, F.; Wittmann, D.; Pindl, S.; Wagner, S.; Gahoi, A.; Reato, E.; Belete, M.; Kataria, S.; Lemme, M. C. Dielectric Surface Charge Engineering for Electrostatic Doping of Graphene. *ACS Applied Electronic Materials* **2020**, *2*, 1235–1242, DOI: 10.1021/acsaelm.0c00051.

(22) Bolotin, K. I.; Sikes, K. J.; Jiang, Z.; Klima, M.; Fudenberg, G.; Hone, J.; Kim, P.; Stormer, H. L. Ultrahigh electron mobility in suspended graphene. *Solid State Communications* **2008**, *146*, 351–355, DOI: 10.1016/j.ssc.2008.02.024.

(23) Ma, N.; Jena, D. Carrier statistics and quantum capacitance effects on mobility extraction in two-dimensional crystal semiconductor field-effect transistors. *2D Materials* **2015**, *2*, 015003, DOI: 10.1088/2053-1583/2/1/015003.

(24) Xia, J.; Chen, F.; Li, J.; Tao, N. Measurement of the quantum capacitance of graphene. *Nature Nanotechnology* **2009**, *4*, 505–509, DOI: 10.1038/nnano.2009.177.

(25) Zhang, Y.; Tan, Y. W.; Stormer, H. L.; Kim, P. Experimental observation of the quantum Hall effect and Berry's phase in graphene. *Nature* **2005**, *438*, 201–204, DOI: 10.1038/nature04235.

(26) Zhang, Y.; Brar, V. W.; Wang, F.; Girit, C.; Yayon, Y.; Panlasigui, M.; Zettl, A.; Crommie, M. F. Giant phonon-induced conductance in scanning tunnelling spectroscopy of gate-tunable graphene. *Nature Physics* **2008**, *4*, 627–630, DOI: 10.1038/nphys1022.

(27) Park, J.; Lee, W. H.; Huh, S.; Sim, S. H.; Kim, S. B.; Cho, K.; Hong, B. H.; Kim, K. S. Work-function engineering of graphene electrodes by self-assembled monolayers for high-performance organic field-effect transistors. *Journal of Physical Chemistry Letters* **2011**, *2*, 841–845, DOI: 10.1021/jz200265w.

(28) Martin, J.; Akerman, N.; Ulbricht, G.; Lohmann, T.; Smet, J. H.; Von Klitzing, K.; Yacoby, A. Observation of electron-hole puddles in graphene using a scanning single-electron transistor. *Nature Physics* **2008**, *4*, 144–148, DOI: 10.1038/nphys781.

(29) Suk, J. W.; Kitt, A.; Magnuson, C. W.; Hao, Y.; Ahmed, S.; An, J.; Swan, A. K.; Goldberg, B. B.; Ruoff, R. S. Transfer of CVD-grown monolayer graphene onto arbitrary substrates. *ACS Nano* **2011**, *5*, 6916–6924, DOI: 10.1021/nn201207c.

(30) Sinterhauf, A.; Traeger, G. A.; Momeni Pakdehi, D.; Schädlich, P.; Willke, P.; Speck, F.; Seyller, T.; Tegenkamp, C.; Pierz, K.; Schumacher, H. W.; Wenderoth, M. Substrate induced nanoscale resistance variation in epitaxial graphene. *Nature Communications* **2020**, *11*, 1–9, DOI: 10.1038/s41467-019-14192-0.

(31) Rhodes, D.; Chae, S. H.; Ribeiro-Palau, R.; Hone, J. Disorder in van der Waals heterostructures of 2D materials. *Nature Materials* **2019**, *18*, 541–549, DOI: 10.1038/s41563-019-0366-8.

(32) Degraeve, R.; Cho, M.; Govoreanu, B.; Kaczer, B.; Zahid, M. B.; Van Houdt, J.; Jurczak, M.; Groeseneken, G. Trap spectroscopy by charge injection and sensing (TSCIS). *International Electron Devices Meeting, IEDM* **2008**, 10–13, DOI: 10.1109/IEDM.2008.4796812.

(33) Zahid, M. B.; Aguado, D. R.; Degraeve, R.; Wang, W. C.; Govoreanu, B.; Toledano-Luque, M.; Afanas'Ev, V. V.; Van Houdt, J. Applying complementary trap characterization technique to crystalline γ-phase-Al_2O_3. *IEEE Transactions on Electron Devices* **2010**, *57*, 2907–2916, DOI: 10.1109/TED.2010.2071071.

(34) Franco, J. et al. Suitability of high-k gate oxides for III-V devices: A PBTI study in $In_{0.53}Ga_{0.47}As$ devices with Al_2O_3. *IEEE International Reliability Physics Symposium Proceedings* **2014**, 6–11, DOI: 10.1109/IRPS.2014.6861098.

(35) Putcha, V.; Franco, J.; Vais, A.; Kaczer, B.; Sioncke, S.; Linten, D.; Groeseneken, G. Impact of slow and fast oxide traps on $In_{0.53}Ga_{0.47}As$ device operation studied using CET maps. *IEEE International Reliability Physics Symposium Proceedings* **2018**, 5A.31–5A.37, DOI: 10.1109/IRPS.2018.8353603.

(36) Illarionov, Y.; Knobloch, T.; Waltl, M.; Rzepa, G.; Pospischil, A.; Polyushkin, D.; Furchi, M.; Mueller, T.; Grasser, T. Energetic mapping of oxide traps in MoS2 field-effect transistors. *2D Materials* **2017**, *4*, 025108, DOI: 10.1088/2053-1583/aa734a.

(37) Guo, Y.; Li, H.; Robertson, J. AlN and Al oxy-nitride gate dielectrics for reliable gate stacks on Ge and InGaAs channels. *Journal of Applied Physics* **2016**, *119*, DOI: 10.1063/1.4951004.

(38) Dicks, O. A.; Cottom, J.; Shluger, A. L.; Afanas'Ev, V. V. The Origin of Negative Charging in Amorphous Al_2O_3 Films: The Role of Native Defects. *Nanotechnology* **2019**, *30*, DOI: 10.1088/1361-6528/ab0450.

(39) IEEE, *IRDS More Moore*; 2020; pp 1–30.

(40) Wei, W.; Pallecchi, E.; Haque, S.; Borini, S.; Avramovic, V.; Centeno, A.; Amaia, Z.; Happy, H. Mechanically robust 39 GHz cut-off frequency graphene field effect transistors on flexible substrates. *Nanoscale* **2016**, *8*, 14097–14103, DOI: 10.1039/c6nr01521b.

(41) Bonmann, M.; Asad, M.; Yang, X.; Generalov, A.; Vorobiev, A.; Banszerus, L.; Stampfer, C.; Otto, M.; Neumaier, D.; Stake, J. Graphene field-effect transistors with high extrinsic f_T and f_{max}. *IEEE Electron Device Letters* **2019**, *40*, 131–134, DOI: 10.1109/LED.2018.2884054.

(42) Kedzierski, J.; Hsu, P. L.; Reina, A.; Kong, J.; Healey, P.; Wyatt, P.; Keast, C. Graphene-on-insulator transistors made using C on Ni chemical-vapor deposition. *IEEE Electron Device Letters* **2009**, *30*, 745–747, DOI: 10.1109/LED.2009.2020615.

(43) Lemme, M. C.; Member, S.; Echtermeyer, T. J.; Baus, M.; Kurz, H. A Graphene Field-Effect Device. *IEEE Electron Device Letters* **2007**, *28*, 282–284.

(44) Uzlu, B.; Wang, Z.; Lukas, S.; Otto, M.; Lemme, M. C.; Neumaier, D. Gate-tunable graphene-based Hall sensors on flexible substrates with increased sensitivity. *Scientific Reports* **2019**, 1–7, DOI: 10.1038/s41598-019-54489-0.

(45) Mueller, T.; Xia, F.; Avouris, P. Graphene photodetectors for high-speed optical communications. *Nature Photonics* **2010**, *4*, 297–301, DOI: 10.1038/nphoton.2010.40.

(46) Konstantatos, G.; Badioli, M.; Gaudreau, L.; Osmond, J.; Bernechea, M.; De Arquer, F.; Gatti, F.; Koppens, F. Hybrid Graphene–Quantum Dot Phototransistors with Ultrahigh Gain. *Nat. Nanotechnol.* **2012**, *7*, 363.

(47) Bartolomeo, A. D.; Genovese, L.; Giubileo, F.; Iemmo, L.; Luongo, G.; Foller, T.; Schleberger, M. Hysteresis in the transfer characteristics of MoS2 transistors. *2D Materials* **2018**, *5*, 1–9, DOI: 10.1088/2053-1583/aa91a7.

(48) Deal, B. E.; Sklar, M.; Grove, a. S.; Snow, E. H. Characteristics of the Surface-State Charge (Qss) of Thermally Oxidized Silicon. *Journal of The Electrochemical Society* **1967**, *114*, 266, DOI: 10.1149/1.2426565.

(49) Stathis, J. H.; Zafar, S. The negative bias temperature instability in MOS devices: A review. *Microelectronics Reliability* **2006**, *46*, 270–286, DOI: 10.1016/j.microrel.2005.08.001.

(50) Grasser, T.; Kaczer, B.; Goes, W.; Reisinger, H.; Aichinger, T.; Hehenberger, P.; Wagner, P.-j.; Schanovsky, F.; Franco, J.; Luque, M. T.; Nelhiebel, M. The Paradigm Shift in Understanding the Bias Temperature Instability: From Reaction-Diffusion to Switching Oxide Traps. *Transactions on Device and Materials Reliability, IEEE* **2011**, *58*, 3652–3666, DOI: 10.1109/TED.2011.2164543.

(51) Illarionov, Y. Y.; Smith, A. D.; Vaziri, S.; Ostling, M.; Mueller, T.; Lemme, M. C.; Grasser, T. Bias-temperature instability in single-layer graphene field-effect transistors. *Applied Physics Letters* **2014**, *105*, DOI: 10.1063/1.4897344.

(52) Illarionov, Y.; Waltl, M.; Smith, A.; Vaziri, S.; Ostling, M.; Lemme, M.; Grasser, T. Interplay between hot carrier and bias stress components in single-layer double-gated graphene field-effect transistors. European Solid-State Device Research Conference. 2015; pp 172–175, DOI: 10.1109/ESSDERC.2015.7324741.

(53) Illarionov, Y.; Molina-Mendoza, A.; Waltl, M.; Knobloch, T.; Furchi, M.; Mueller, T.; Grasser, T. Reliability of next-generation field-effect transistors with transition metal dichalcogenides. IEEE International Reliability Physics Symposium Proceedings. 2018; DOI: 10.1109/IRPS.2018.8353605.

(54) Illarionov, Y. Y.; Knobloch, T.; Smithe, K. K. H.; Waltl, M.; Grady, R. W.; Waldhoer, D.; Pop, E.; Grasser, T. Anomalous Instabilities in CVD-MoS_2 FETs Suppressed by High-Quality Al2O3 Encapsulation. Device Research Conference - Conference Digest, DRC. Columbus, OH, 2020; pp 150–151.
